# *Lactobacillus salivarius* Probiotic Supplementation Modulates Gut Function, Improves Growth, and Meat Quality in Tropical Whiteleg Shrimp

**DOI:** 10.1155/anu/6285997

**Published:** 2026-05-27

**Authors:** Farhana Najnine, Xinbo Guo, Junpeng Cai

**Affiliations:** ^1^ School of Food Science and Engineering, South China University of Technology, Guangzhou, 510641, China, scut.edu.cn

**Keywords:** 4D-DIA proteomics, gut microbiota, high-throughput sequencing, *Lactobacillus salivarius*, *Litopenaeus vannamei*, meat quality, probiotic, tropical aquaculture

## Abstract

Probiotics are increasingly incorporated into shrimp aquaculture, yet the comparative efficacy of single‑strain versus mixed‑strain formulations remains insufficiently defined, particularly in relation to gut microbiome remodeling, intestinal architecture, and proteomic determinants of meat quality. A 90‑day randomized grow‑out trial was conducted under semi‑intensive conditions using *Litopenaeus vannamei* fed three diets (triplicate ponds per treatment): basal diet (W), basal diet plus mixed‑strain EM probiotic (T), and basal diet plus *Lactobacillus salivarius* GZPH2 (H). Growth performance, survival, intestinal histology, microbiota composition and function (16S/18S rRNA sequencing), and meat quality (4D‑DIA proteomics) were evaluated. Both probiotics improved growth and feed utilization relative to controls, but H consistently outperformed T in survival (83.0% vs. 81.0%), meat yield (6.53% vs. 4.84%), lipid deposition (4.05% vs. 3.39%), and mineral enrichment (Fe, Se; Zn/Cu ratio: 2.27 vs. 1.97). H enhanced microbial α‑diversity and enriched metabolically versatile taxa (*Achromobacter*, *Pedobacter*, *Photobacterium*, and *Labrenzia*), whereas T produced *Bacillus*‑dominant communities (~85%) with reduced diversity. Intestinal morphology showed greater absorptive expansion in H (+14.10%) and higher goblet cell density in T (+51.21%). Functional profiling indicated that H activated KEGG pathways related to amino acid transport, nitrogen metabolism, fatty acid biosynthesis, and trace‑mineral transport, supporting enhanced protein turnover, lipid deposition, mineral assimilation, and redox‑homeostasis. In contrast, T favored carbohydrate metabolism and antimicrobial gene expression, promoting competitive exclusion but limiting metabolic versatility. Proteomic analysis identified 183 differentially expressed proteins (DEPs, 110 upregulated in H; 73 in T). H upregulated myofibrillar and antioxidant proteins, including myosin heavy chain type 2, hemocyanin, catalase, metallothionein, and selenium‑binding protein, while suppressing proteolytic and glycolytic enzymes, thereby improving texture, oxidative stability, and nutritional value of meat. Collectively, *Lactobacillus salivarius* GZPH2 demonstrated superior capacity for metabolic modulation, intestinal remodeling, and proteomic enhancement, highlighting its potential as a targeted probiotic for optimizing shrimp health and meat quality in aquaculture nutrition.

## 1. Introduction

The sustainability of global shrimp aquaculture, a cornerstone of the blue economy and global food security, is increasingly constrained by interacting biological, environmental, and economic pressures. The industry, dominated by the Pacific whiteleg shrimp *Litopenaeus vannamei* (*L. vannamei*), supports livelihoods and provides a major source of animal protein worldwide, yet its expansion is limited by escalating feed costs, environmental stress, and recurrent disease outbreaks [1]. These constraints are particularly severe in tropical production systems, where elevated water temperatures increase metabolic demand, disrupt intestinal homeostasis, and compromise immune competence, collectively reducing feed efficiency, growth performance, and farm profitability [2–4]. Simultaneously, market expectations increasingly emphasize not only production efficiency but also consistent product quality with enhanced nutritional and technological attributes. Addressing these challenges requires nutritional strategies that extend beyond growth promotion to strengthen intestinal resilience, physiological stability, and post‐harvest value.

Within this framework, dietary probiotics have therefore emerged as a promising alternative to prophylactic antibiotics in shrimp aquaculture. A substantial body of evidence demonstrates that probiotics can improve growth performance, immune responses, and survival in penaeid shrimp through multiple mechanisms, including modulation of intestinal microbial communities, enhancement of digestive enzyme activity, reinforcement of epithelial barrier integrity, and mitigation of oxidative stress [5–7]. Importantly, probiotic effects extend beyond zootechnical parameters to the final product. Studies in aquatic and terrestrial animals indicate that probiotics can influence post‐harvest meat quality by modifying muscle composition, water‐holding capacity, texture, and oxidative stability, thereby establishing a functional link between gut health and final product quality [8, 9].

Despite these benefits, a critical contradiction exists in current probiotic practice. Most commercial shrimp probiotics are formulated as multi‐strain or consortium‐based products, commonly combining *Bacillus* spp., lactic acid bacteria, photosynthetic bacteria, and yeast [10]. These formulations are generally justified by the assumption that increased microbial diversity confers functional robustness across variable culture conditions. However, accumulating evidence from both aquatic and terrestrial systems challenges this premise. Mixed‐strain probiotics may exhibit inconsistent efficacy due to inter‐strain antagonism, competitive exclusion, or functional redundancy, particularly under environmental stressors such as elevated temperature [10, 11]. Such interactions can impair colonization efficiency and lead to variable host responses across production cycles, undermining the predictability required for commercial application [12]. These observations highlight the need for mechanistic comparisons between complex probiotic consortia and well‐defined single‐strain probiotics selected for specific functional traits and environmental compatibility.

Lactic acid bacteria, including *Lactobacillus*, are widely recognized for their metabolic versatility and probiotic efficacy in aquaculture. Notably, *Lactobacillus salivarius* (*L. salivarius*) is a well‐characterized probiotic in terrestrial livestock, where it exhibits immunomodulatory, antimicrobial, and gut health–promoting properties [13]. However, this strain remains poorly studied in aquaculture. Recently, Najnine et al. [14] investigated *L. salivarius* GZPH2 and reported its effects on hepatopancreatic morphology, microbiome, and protein structure, as well as its role in hepatopancreatic health. Nevertheless, the effects of this strain on intestinal morphology, microbiome, and meat quality remain unclear. This gap is significant, as the success of feed‐based probiotics depends fundamentally on their ability to colonize and beneficially modulate the intestinal environment, thereby eliciting systemic physiological effects mediated through the intestinal microbiota axis [15].

Recent methodological advances enable a more integrated evaluation of probiotic function. High‐throughput sequencing of 16S/18S rRNA genes provides culture‐independent characterization of intestinal microbial community structure and predicted metabolic potential, clarifying the microbiome’s role in host nutrition, metabolism, and physiological homeostasis [16, 17]. In parallel, post‐harvest quality assessment a key determinant of shrimp market value has benefited from advances in proteomic technologies. Shrimp muscle is highly perishable, with quality deterioration driven by proteolytic degradation and lipid oxidation following harvest [18, 19]. Data‐independent acquisition (DIA) mass spectrometry has emerged as a powerful tool in meat science, enabling high‐throughput identification of protein biomarkers associated with freshness, texture, oxidative stability, and spoilage dynamics [20, 21]. However, the application of advanced proteomics to determine how in vivo probiotic supplementation shapes the shrimp muscle proteome and modulates early post‐mortem biochemical pathways remains unexplored.

A critical knowledge gap remains between empirical probiotic use and the mechanistic links among gut microbiota modulation, muscle biochemistry, and shrimp meat quality. To date, no study has integrated growth performance, intestinal histomorphology, microbial ecology, and meat proteomics to directly compare single‐strain *L. salivarius* with multi‐strain probiotics in tropical shrimp aquaculture. We hypothesized that a targeted single‐strain probiotic would induce more beneficial intestinal microbiome modulation and superior meat quality in *L. vannamei* than a commercial multi‐strain effective microorganism (EM) consortium. Accordingly, this study evaluated growth performance and intestinal histomorphology, characterized gut microbiota using 16S/18S rRNA sequencing, and identified meat quality related protein biomarkers using four‐dimensional DIA (4D‐DIA) proteomics. This integrated approach provides the first mechanistic assessment of *L. salivarius* in tropical shrimp aquaculture and supports precision probiotic strategies for sustainable production.

## 2. Materials and Methods

### 2.1. Experimental Design, Shrimp Culture, and Sampling

A 90‐day grow‐out trial was conducted from late April to late July 2023 at a semi‐intensive aquaculture facility in Hainan, China. The region is characterized by a tropical monsoon climate with elevated summer temperatures (30–33°C) and variable precipitation (Supporting Information 1: Figure S1). These conditions provided a representative commercial setting for evaluating probiotic efficacy under natural farming environments.

The experiment followed a completely randomized design with three dietary treatments: (i) control (W), receiving the basal diet only (ponds W1–W3); (ii) treatment T, receiving the basal diet supplemented with a commercial mixed‐strain effective microorganisms (EM) probiotic (ponds T1–T3); and (iii) treatment H, receiving the basal diet supplemented with *Lactobacillus salivarius* GZPH2 (ponds H1–H3). Each treatment was conducted in triplicate (*n* = 9 ponds total) using HDPE‐lined earthen ponds (60 m^2^), each equipped with independent aeration and hydraulically isolated water supply and drainage systems to prevent cross‐contamination (Supporting Information 1: Figure S2). Detailed pond configuration, management practices, and water quality dynamics are provided in Supporting Information 1: Figures S2–S4 and Supporting Information 2: Note S1.

Post‐larval Pacific white shrimp (*L. vannamei*, PL10) were stocked at a density of 120 individuals m^−2^ (7200 shrimp per pond) and cultured under semi‐intensive conditions for 90 days. Shrimp were fed a commercial high‐protein diet (Guangdong Yuehai Feed Group Co., China; proximate composition in Supporting Information 3: Table S1) three times daily following standard aquaculture practices. Probiotic sources, preparation, and feed incorporation methods are described in Supporting Information 2: Note S2. Feeding rates were adjusted weekly based on estimated biomass and feed tray observations, starting at 8%–10% of biomass during early growth and gradually decreasing to 3%–5% as shrimp increased in size.

Standing biomass was estimated weekly using a spatially randomized sampling approach to minimize within‐pond variability. Shrimp were collected from three randomly selected locations per pond using a cast net, and 50 individuals were randomly subsampled and weighed individually (±0.01 g) to determine mean body weight (MBW) (Supporting Information 1: Figure S5). After measurement, shrimp were returned immediately to minimize handling stress. Final biomass and survival were determined by complete harvest, enabling accurate calculation of feed conversion ratio (FCR) (Supporting Information 2: Note S3).

At the end of the culture period, live shrimp were transported to the laboratory for subsequent analyses. No disease outbreaks were observed during the trial. From each pond, 25 visually healthy and undamaged individuals were randomly selected and placed in polyethylene bags containing oxygenated water (dissolved oxygen ≈ 5 mg L^−1^). Samples were transported in insulated, temperature‐controlled containers (<72 h transit). Upon arrival, shrimp vitality was confirmed based on active gill movement and normal responses to tactile stimulation, ensuring suitability for further analyses.

### 2.2. Measurement of Growth Parameters and Yield

**Figure 1:**
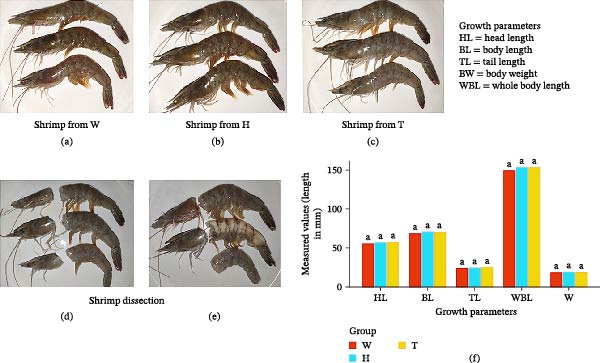
Morphological analysis and growth parameters of shrimp from three experimental groups (W, H, and T). (a–c) Representative images of shrimp from groups W, H, and T, respectively. (d, e) Dissection views of representative shrimp. (f) Comparison of average growth parameters across all groups: head length (HL), body length (BL), tail length (TL), whole‐body length (WBL), and body width (BW). Different lowercase letters above bars indicate statistically significant differences (*p* < 0.05), while the same letters indicate no significant difference (*p* > 0.05). The *x*‐axis represents the growth parameters, and the *y*‐axis indicates the measured values (length in cm).

After harvest all shrimp at the end of experiment to assess growth performance and yield traits at the farm 50 shrimp were randomly selected from per replicate of each treatment group. Shrimp were anesthetized on ice, and surface moisture was removed using sterile filter paper. Morphometric parameters, including head length, abdominal length, tail length, total body length, and second abdominal segment width, were measured using electronic Vernier calipers (precision: 0.1 mm). Whole‐body weight was recorded using an analytical balance (precision: 0.001 g). Each shrimp was dissected into head and abdominal portions (Figure 1d), and weights were recorded separately. The abdominal shell (with tail) was separated from muscle tissue (Figure 1e), and meat and shell weights were measured individually. Total by‐product weight was calculated as the combined weight of the head and abdominal shell with tail. Growth performance and yield parameters were calculated according to the formulas provided in Supporting Information 2: Note S3.

### 2.3. Shrimp Sampling and Preparation of Intestinal and Meat Samples

For intestinal analysis, three live shrimp were randomly selected from each replicate (i.e., each pond) of all treatment groups following transport to the laboratory. Shrimp surfaces were sterilized with 75% ethanol and subsequently rinsed with sterile distilled water to remove external contaminants. The intestines were aseptically dissected, placed on sterile glass slides, and their lengths were measured using electronic Vernier calipers (precision: 0.1 mm). For each replicate, intestines from shrimp were pooled and homogenized in 1 mL sterile water, followed by the addition of 1 mL Tris–EDTA (TE) buffer (pH 7.3–7.5) for subsequent 16S and 18S rRNA gene sequencing. Samples were stored at −20°C until further analysis. Muscle tissues were similarly collected, pooled, and homogenized in phosphate‐buffered saline (1 × PBS, pH 7.3–7.5), and stored at −80°C for downstream proteomic analysis.

### 2.4. Histological Analysis of Shrimp Intestine

Three adult *L. vannamei* individuals per replicate from each treatment were euthanized by thermal shock. Intestinal tissues were carefully dissected and immediately fixed in Bouin’s solution for 24 h. Samples were coded according to treatment name, tissue type, and replicate number as follows: WI1–WI3, HI1–HI3, and TI1–TI3. Histological analysis of shrimp intestine was performed following a work‐flow similar to that described by Najnine et al. [14]. Histological images were acquired using a Nikon Eclipse Ci light microscope equipped with a DS‐U3 digital camera system. For histomorphometric analysis, images of the intestine were captured and analyzed using SlideViewer software (v2.5). For each treatment, nine well‐oriented micrographs (100× magnification) with intact morphology were selected to minimize processing artifacts. Quantitative parameters included total mucosal thickness (TMU), thickness of the muscularis externa (TME), and thicknesses of the circular (TCM) and longitudinal (TLM) muscle layers. As the shrimp intestine lacks prominent villi or microvilli, the intestinal absorption area (A) was estimated as:

A=P×L.

Here, the formula is derived from basic geometric and physiological principles used to estimate the mucosal surface area of smooth‐walled tubular organs. *A* is the total intestinal absorption area, perimeter (*P*) defines the circumference of the irregular lumen, *L* is the longitudinal stretch of the intestine.

Where *P* represents the perimeter of the elliptical lumen, approximated using Ramanujan’s formula:

P= π 3 a+b−3a+ba+3b .

Here, π = 3.14, *a* is the semi‐major axis, and *b* is the semi‐minor axis.

### 2.5. High‐Throughput Sequencing of Intestinal Microbial Communities

Intestinal homogenates, prepared as described above (shrimp sampling and preparation of intestinal and meat samples) were used for microbial community profiling via high‐throughput sequencing of the prokaryotic 16S rRNA gene and the eukaryotic 18S rRNA gene. Sequencing was performed by Shanghai Majorbio Bio‐Pharm Technology Co., Ltd. (Shanghai, China), following a work‐flow similar to that described by Najnine et al. [14].

Briefly, total genomic DNA was extracted from intestinal homogenates using the FAST DNA Spin Kit (MP Biomedicals, USA) according to the manufacturer’s instructions, with minor modifications to enhance yield. DNA concentration and purity were assessed using a NanoDrop 2000 spectrophotometer (Thermo Fisher Scientific, USA), and DNA integrity was confirmed by 1% agarose gel electrophoresis. Only high‐quality DNA samples proceeded to amplification and sequencing. PCR amplification of the 16S and 18S rRNA genes was carried out on an ABI GeneAmp 9700 thermal cycler (Applied Biosystems, USA). The V3–V4 region of the bacterial 16S rRNA gene was amplified using primers 338F (5′‐ACTCCTACGGGAGGCAGCAG‐3′) and 806R (5′‐GGACTACHVGGGTWTCTAAT‐3′). The V4 region of the eukaryotic 18S rRNA gene was amplified using primers 3NDF (5′‐GGCAAGTCTGGTGCCAG‐3′) and V4‐euk‐R2 (5′‐ACGGTATCTRATCRTCTTCG‐3′). PCR products were purified with the QIAquick PCR Purification Kit (Qiagen, Germany). Paired‐end sequencing (2 × 300 bp) was performed on an Illumina MiSeq platform. Raw reads were quality‐filtered, merged using FLASH, and checked for chimeras with UCHIME. Operational taxonomic units (OTUs) were clustered at 97% similarity using UCLUST. Taxonomic assignment was performed against the SILVA database (release 138). Rarefied datasets were used to calculate alpha and beta diversity indices in QIIME (v1.9.1). Raw sequencing data have been deposited in the NCBI Sequence Read Archive (SRA) under BioProject accession PRJNA1158989, including runs SRR30631632, SRR30631634, SRR30631636, SRR30631644, SRR30631658, SRR30631660, SRR30631662, SRR30631664, and SRR30631666.

### 2.6. Nutritional Composition Analysis of Shrimp Meat

For each replicate per treatment, three healthy shrimp were randomly selected. Each shrimp was carefully peeled, the meat was removed, and the intestine was extracted. The meat from all three shrimp was then crushed and thoroughly mixed using a sterile mortar. From this pooled sample, ~2 g of meat was taken per replicate for each analysis: crude protein, crude lipid, ash content, and moisture content.

The proximate composition of shrimp meat, including crude protein, crude lipid, moisture, and ash contents, was determined following standard methods of the Association of Official Analytical Chemists (AOAC). For each replicate per treatment, ~2 g of meat was taken from three healthy shrimp randomly selected from that replicate. Crude protein content was quantified using the Kjeldahl method (AOAC 928.08) with a Hanon K9860 Automatic Kjeldahl Analyzer (Hanon Advanced Technology Group Co., Ltd., China), applying a nitrogen‐to‐protein conversion factor of 6.25. Crude lipids were measured by Soxhlet extraction (AOAC 991.36) using a Soxtec‐8000 system (Foss, Denmark). Ash content was determined by incineration in a muffle furnace at 550°C for 24 h (AOAC 920.153). Moisture content was determined by oven‐drying samples at 105°C until a constant weight was achieved, and the moisture percentage was calculated based on weight loss using the following formula:

Moisture content %=WInitial−WFinal×100WInitial,

where *W*_Initial_ is the initial weight of the sample before drying (g). *W*_Final_ is the final weight of the sample after drying (g).

Mineral content was analyzed in triplicate for each sample group using inductively coupled plasma optical emission spectrometry (ICP‐OES; iCAP 7400, Thermo Fisher Scientific, USA), following a work‐flow similar to that described by Najnine et al. [14]. with minor modifications. Briefly, ~5 g of meat sample taken from the same pooled samples was digested in a polytetrafluoroethylene (PTFE) tube (Ningbo Ja‐Hely Technology Co., Ltd., China) with 12 mL of concentrated nitric acid (68%, Shijiazhuang Famechem Chemical Co., Ltd., China) until a clear solution was obtained. After cooling, the digestate was diluted to 50 mL with deionized water in a volumetric flask. A blank sample was included for quality control.

To measure pH changes during postmortem storage, shrimp meat pH was determined using a digital pH meter (HI 2211 pH Meter, Hanna Instruments, USA). Details of the pH measurement procedure for shrimp meat are provided in Supporting Information 2: Note S4.

### 2.7. Meat Color Measurement (Raw and Boiled)

Shrimp meat color was evaluated using a portable colorimeter (Minolta CR‑400/CR‑410, Konica Minolta, Japan) operating under the CIELAB system. Details of the color measurement procedure for raw and boiled shrimp meat are provided in Supporting Information 2: Note S5. The parameters measured included lightness (*L*  ^∗^), redness (*a*  ^∗^), and yellowness (*b*  ^∗^) for both raw and boiled samples. The total color difference (Δ*E*) between raw and cooked meat was calculated to quantify the magnitude of color change during thermal processing using the standard CIELAB equation [22]:

ΔE=Lraw∗−Lboiled∗2+araw∗−aboiled∗2+braw∗−bboiled∗2.

This parameter reflects the overall perceptible color shift resulting from cooking.

### 2.8. 4D‐DIA Proteomic Profiling of Shrimp Meat

Muscle tissue samples prepared as described above were used for proteomic analysis. Proteomic variation among shrimp meat samples was examined using a 4D‐DIA mass spectrometry approach. All procedures, including sample preparation, LC–MS/MS analysis, and primary data processing, were performed by Shanghai Majorbio Bio‐Pharm Technology Co., Ltd. (Shanghai, China) using standardized and validated protocols [23], following a work‐flow similar to that described by Najnine et al. [14].

Briefly, frozen shrimp muscle tissues (prepared as described above in shrimp sampling and preparation of intestinal and meat samples) were thawed on ice and lysed in a denaturing buffer containing 8 M urea and 1% SDS supplemented with protease inhibitors. Samples were homogenized using a high‐speed tissue grinder (three cycles of 40 s), incubated on ice for 30 min with intermittent mixing, and centrifuged at 16,000×*g* for 30 min at 4°C. The supernatants were collected, and protein concentrations were determined using a bicinchoninic acid (BCA) assay (Thermo Fisher Scientific, USA). Protein quality was assessed by SDS–PAGE.

For proteolytic digestion, 100 μg of protein per sample was diluted in triethylammonium bicarbonate (TEAB) buffer, reduced with tris(2‐carboxyethyl)phosphine (10 mM, 37°C for 60 min), and alkylated with iodoacetamide (40 mM, room temperature for 40 min in the dark). Proteins were subsequently digested overnight with sequencing‐grade trypsin (1:50, w/w) at 37°C. The resulting peptides were vacuum‐dried, resuspended in 0.1% trifluoroacetic acid, desalted using hydrophilic–lipophilic balance cartridges, and quantified. Peptide analysis was performed on a timsTOF Pro 2 mass spectrometer (Bruker, Germany) operating in DIA‐PASEF mode, coupled with nano‐LC separation on a C18 column (150 μm × 15 cm) at a flow rate of 500 nL/min using a 30‐step gradient. Data were acquired over an m/z range of 400–1200 with 64 DIA‐PASEF windows. Raw data were processed using Spectronaut (v14) with indexed retention time calibration. A false discovery rate (FDR) of ≤1% was applied at both peptide and protein levels. Only proteins identified with at least one unique peptide were retained for subsequent analyses.

### 2.9. Data Analysis

#### 2.9.1. Statistical Analysis

All statistical analyses were performed using SPSS (v26.0). One‐way analysis of variance (ANOVA) followed by Tukey’s post‐hoc test was applied to determine significant differences among experimental groups (W, H, T) at *p* < 0.05. Pearson’s correlation coefficients (*r*) were calculated to assess relationships between variables. Data were presented in tables, and visualizations were generated using R (v4.3.2).

#### 2.9.2. Microbial Diversity and Community Analysis

Amplicon sequence variant (ASV) tables were used to evaluate microbial communities. Alpha diversity indices, including sequencing depth (coverage), ACE, Chao1, Shannon, and Simpson were computed using Mothur (v1.30.2) to quantify taxonomic richness and diversity. Beta diversity was assessed via principal coordinate analysis (PCoA) based on ASV‐level distance matrices, with significance tested using ANOSIM (999 permutations) in R (vegan package, v3.3.1). Community composition was visualized at multiple taxonomic levels (phylum, class, genus) using bar plots, pie charts, and Venn diagrams to highlight unique and shared taxa across groups. Differential abundance was tested using the Kruskal–Wallis H test with Tukey–Kramer post‐hoc correction, and discriminative taxa were identified via linear discriminant analysis effect size (LEfSe) using the LEfSe Galaxy platform. Co‐occurrence networks were constructed with the R igraph package (v3.2) using the Kamada‐Kawai layout, focusing on the top 50 abundant genera and Spearman correlations (*p* < 0.05).

#### 2.9.3. Functional Prediction

Microbial functional traits were predicted using BugBase (https://bugbase.cs.umn.edu), normalizing ASVs by 16S rRNA copy number to assess phenotypes such as Gram status, biofilm formation, pathogenicity, oxygen utilization, and stress tolerance. Metabolic potential was inferred with phylogenetic investigation of communities by reconstruction of unobserved states (PICRUSt; v1.0.0), linking ASVs to Clusters of Orthologous Groups (COG), Kyoto Encyclopedia of Genes and Genomes (KEGG) orthologues (KO), and MetaCyc pathways. Functional annotations were further refined using evolutionary genealogy of genes: non‐supervised orthologous groups (eggNOG) database classifications, whereas KO and MetaCyc pathway abundances were mapped onto KEGG hierarchies. PICRUSt further generated stratified metabolic pathway abundances across three functional tiers (pathway, module, and reaction levels) to resolve the ecosystem‐scale metabolic potential.

#### 2.9.4. Proteomic Analysis

Proteomic data were processed via the Majorbio Cloud Platform (https://cloud.majorbio.com). Protein sequences were annotated against KEGG, EggNOG, Gene Ontology (GO), Pfam, and subcellular localization databases. Differentially expressed proteins (DEPs) were defined by |log_2_FC| ≥ 0.263 and *p* < 0.05. Functional and pathway enrichment analyses were conducted using GO, KEGG, and EggNOG, with significance determined by Fisher’s exact test and Benjamini–Hochberg FDR correction (FDR < 0.05).

## 3. Results

### 3.1. Comparative Effects of Probiotic Treatments on Growth Performance and Yield

Both probiotic treatments (H and T) significantly enhanced growth performance and yield parameters compared to the control (Table 1; Figure 1). Overall, the two probiotic groups exhibited comparable efficacy, with only minor differences observed between them. Compared to the mixed probiotic treatment (T), the single‐strain treatment (H) demonstrated superior advantages in survival rate (83.0% vs. 81.0%) and meat yield (6.53% vs. 4.84%). In addition, the meat‐to‐waste ratio was marginally greater in H (1.49 ± 0.05) compared to T (1.47 ± 0.08), reflecting a small advantage in edible yield efficiency.

**Table 1:** Growth performance, body composition, yield, survival, and feed utilization of shrimp under different treatments.

Parameter	W	H	T
Initial body weight (g)	0.100 ± 0.01	0.103 ± 0.01	0.100 ± 0.01
Final body weight (g)	21.32 ± 1.15^b^	24.41 ± 0.62^a^	24.85 ± 0.22^a^
Head weight (g)	8.20 ± 0.34^a^	8.82 ± 0.06^a^	8.97 ± 0.13^a^
Body weight (g)	13.12 ± 0.89^b^	15.84 ± 0.64^a^	15.88 ± 0.78^a^
Meat weight (g)	12.11 ± 0.82^b^	14.77 ± 0.59^a^	14.80 ± 0.62^a^
Abdominal shell with tail weight (g)	1.01 ± 0.06^a^	1.07 ± 0.04^a^	1.08 ± 0.06^a^
Total by‐product (g)	9.20 ± 0.04^a^	9.89 ± 0.08^a^	10.05 ± 0.07^a^
Meat yield (g/100 g)	56.80 ± 1.04^b^	60.51 ± 0.92^a^	59.55 ± 1.43^a^
By‐product yield (g/100 g)	43.19 ± 1.03^a^	40.52 ± 1.92^b^	40.44 ± 1.43^a^
Meat : waste ratio	1.31 ± 0.05^b^	1.49 ± 0.05^a^	1.47 ± 0.08^a^
Total weight gain (g)	21.23 ± 1.15^b^	24.31 ± 0.62^a^	24.75 ± 0.22^a^
Average daily gain (ADG, g day^−1^)	0.236 ± 0.013^b^	0.270 ± 0.007^a^	0.275 ± 0.002^a^
Specific growth rate (SGR, % day^−1^)	5.96 ± 0.06^b^	6.11 ± 0.03^a^	6.13 ± 0.01^a^
Total biomass gain (kg)	115.26 ± 1.81^b^	145.37 ± 0.71^a^	144.76 ± 0.84^a^
Total feed input (kg)	216.9 ± 2.10^b^	230.4 ± 0.94^a^	228.3 ± 0.96^a^
Feed conversion ratio (FCR)	1.88 ± 0.01^a^	1.59 ± 0.04^b^	1.58 ± 0.03^b^
Survival rate (%)	75.0 ± 1.20^c^	83.0 ± 3.08^a^	81.0 ± 1.90^b^

*Note:* Data are mean ± SD (*n* = 3 replicate ponds per treatment: WS1–WS3, HS1–HS3, TS1–TS3). Different superscript letters in the same column denote statistically significant differences (*p* < 0.05), while identical letters indicate no significant differences (*p* > 0.05).

### 3.2. Intestinal Histomorphological Responses to Probiotic Supplementation

**Figure 2:**
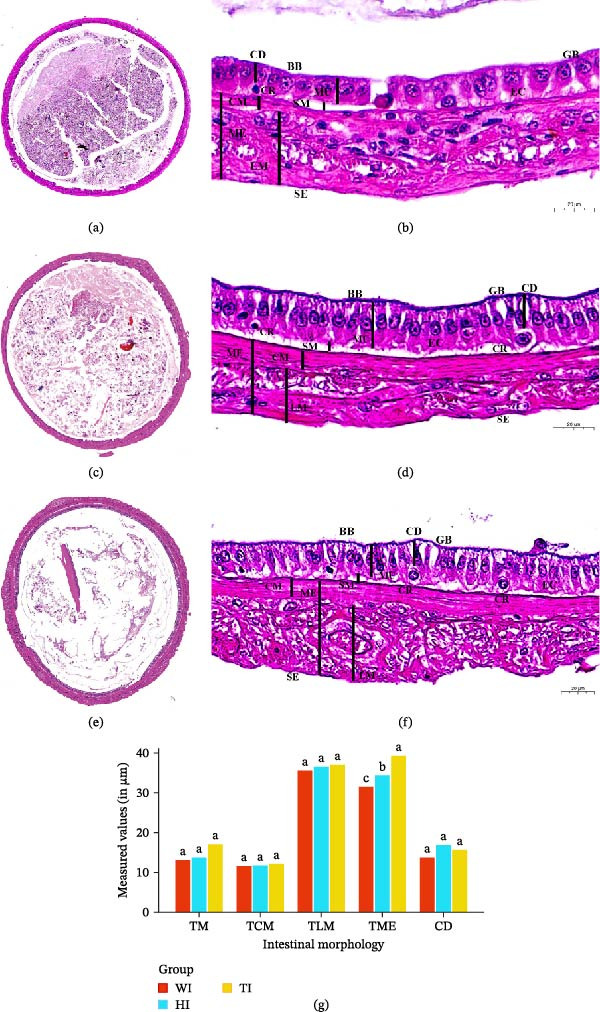
Histological structures of the intestines in the three shrimp groups (W, H, and T). (a–f) Intestinal transverse sections: (a, b) W group; (c, d) H group; (e, f) T group. Key structures: mucosa (MU), submucosa (SM), circular muscle (CM), longitudinal muscle (LM), muscularis externa (ME), crypt (CR), crypt depth (CD), goblet cell (GB), enterocyte (EC), and serosa (SE). (g) Histogram comparing intestinal morphological parameters across groups: mucosal thickness (TM), muscularis externa thickness (TME), circular muscle thickness (TCM), longitudinal muscle thickness (TLM), and crypt depth (CD). Different letters above bars indicate significant differences (*p* < 0.05); shared letters indicate no significant difference (*p* > 0.05). The *x*‐axis represents the intestinal morphological parameters, and the *y*‐axis indicates the measured values (in micrometers, μm).

Histological analysis revealed that shrimp intestines across all treatments retained a typical four‐layered organization (serosa, muscularis externa, submucosa, and mucosa) with a simple columnar epithelium containing abundant enterocytes and goblet cells, indicating preserved intestinal integrity (Figure 2a–f). No significant differences in intestinal length were observed among treatments (*p* > 0.05).

Despite conserved architecture, probiotic supplementation induced significant histomorphological alterations (Table 2). Transverse longitudinal muscle (TLM) thickness increased in probiotic‐treated shrimp (*T* > *H* > *W*; *p* < 0.05). Total absorption area increased by 14.10% in H and 3.28% in T relative to W (*p* < 0.05). Goblet cell density was elevated in both probiotic groups, with greater enhancement in T (51.21%) than H (19.45%) (*p* < 0.05). Enterocyte numbers were significantly increased in H and T compared to W, with no significant difference between the two probiotic treatments.

**Table 2:** The number of different cells of transverse section of intestine in the three groups of shrimp.

Group	Intestinal length (mm)	Total absorption area (mm^2^)	Goblet cell	Enterocytes
WI	66.96 ± 0.8^a^	423.40 ± 5.30^b^	13.67 ± 1.15^b^	18.87 ± 1.18^b^
HI	67.76 ± 0.85^a^	483.12 ± 6.98^a^	16.33 ± 0.57^ab^	32.33 ± 0.77^a^
TI	67.43 ± 0.46^a^	437.29 ± 9.26^ab^	20.67 ± 2.08^a^	31.67 ± 1.08^a^

*Note:* Data are mean ± SD (*n* = 3 replicate ponds per treatment: WS1–WS3, HS1–HS3, TS1–TS3). Different superscript letters in the same column denote statistically significant differences (*p* < 0.05), while identical letters indicate no significant differences (*p* > 0.05). WI, HI, and TI denote the intestinal samples from groups W, H, and T, respectively.

### 3.3. Effects of Probiotics on Intestinal Microbial Diversity

High‐throughput 16S rRNA gene sequencing generated 441,613 high‐quality reads clustered into 635 ASVs, indicating sufficient sequencing depth (Table 3). T showed the highest sequencing depth but the lowest richness and diversity, whereas *H* exhibited significantly higher bacterial richness and diversity despite lower read counts (*p* < 0.05).

**Table 3:** The bacterial diversity and richness indices for each intestine sample in three groups of shrimp.

Group	Sequence number	ASV number	Shannon index	Simpson index	ACE index	Chao1 index	Coverage
W	40996.00 ± 2979.34^a^	77.00 ± 27.87^ab^	0.72 ± 0.26^b^	0.74 ± 0.08^a^	77.99 ± 27.11^ab^	77.18 ± 27.85^ab^	0.99 ± 0^a^
H	32406.33 ± 1463.87^b^	114.00 ± 43.26^a^	2.06 ± 0.3^a^	0.26 ± 0.03^b^	114.05 ± 43.36^a^	114 ± 43.26^a^	0.99 ± 0^a^
T	45409.00 ± 3028.00^a^	31.00 ± 8.00^b^	0.42 ± 0.2^b^	0.76 ± 0.16^a^	30.88 ± 7.38^b^	30.5 ± 7.5^b^	0.99 ± 0^a^

*Note:* Data are mean ± SD (*n* = 3 replicate ponds per treatment: WS1–WS3, HS1–HS3, TS1–TS3). Different superscript letters in the same column denote statistically significant differences (*p* < 0.05), while identical letters indicate no significant differences (*p* > 0.05).

Alpha‐diversity indices supported these patterns. Shannon diversity was significantly higher in H compared to W and T (*p* < 0.05), while Simpson index values indicated stronger dominance in T. Richness estimators (ACE and Chao1) were also higher in H than in W and T (*p* < 0.05). Coverage values (~0.99) confirmed adequate sampling depth.

In contrast, 18S rRNA sequencing yielded 395,744 reads clustered into 32 ASVs, with no significant differences were observed among groups in sequence number, ASV number, Shannon index, ACE index, or Chao1 index (*p* > 0.05; Table 4), these results indicate that probiotic supplementation did not substantially alter intestinal microbial alpha diversity, with only minor differences observed in evenness and coverage.

**Table 4:** Eukaryotic diversity and richness indices relative to each intestine sample in three groups of shrimp.

Group	Sequence number	ASV number	Shannon index	Simpson index	ACE index	Chao1 index	Coverage
W	9581.33 ± 1217.74^a^	3.33 ± 2.30^a^	0.01 ± 0.01^a^	0.99 ± 0.00^a^	2.66 ± 3.05^a^	3.33 ± 2.30^a^	1.00 ± 0.00^a^
H	9676 ± 1627.55^a^	3.33 ± 1.15^a^	0.02 ± 0.02^a^	0.99 ± 0.00^ab^	2.00 ± 2.00^a^	3.33 ± 1.15^a^	1.00 ± 0.00^a^
T	11066.33 ± 1893.50^a^	6.00 ± 2.00^a^	0.06 ± 0.01^a^	0.98 ± 0.00^b^	3.50 ± 3.50^a^	5.5 ± 1.50^a^	0.99 ± 0.00^b^

*Note:* Data are mean ± SD (*n* = 3 replicate ponds per treatment: WS1–WS3, HS1–HS3, TS1–TS3). Different superscript letters in the same column denote statistically significant differences (*p* < 0.05), while identical letters indicate no significant differences (*p* > 0.05).

**Figure 3:**
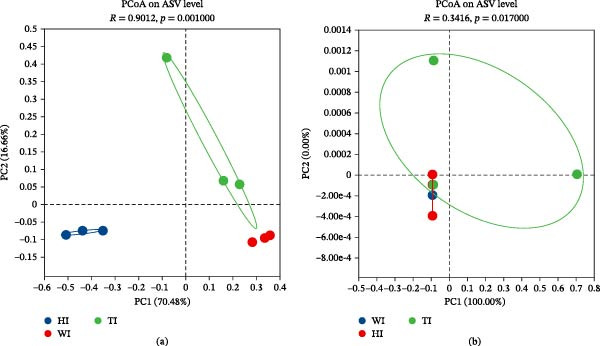
Beta diversity analysis. Principal Coordinate Analysis (PCoA) based on Bray‐Curtis distances was performed to assess microbial community composition. The ordination plots display (a) prokaryotic (16S rRNA) and (b) eukaryotic (18S rRNA) profiles from intestinal samples across three groups (W, H, and T).

Beta diversity analysis further demonstrated clear differences in bacterial community composition. Principal coordinate analysis (PCoA) based on Bray–Curtis dissimilarity showed distinct clustering among treatments (Figure 3a), with strong statistical separation (ANOSIM: *R* = 0.9835, *p* = 0.001). The first two axes explained 80.14% of total variation. In contrast, eukaryotic communities showed weaker separation (ANOSIM: *R* = 0.2140, *p* = 0.05; Figure 3b), indicating limited response to probiotic supplementation.

### 3.4. Effects of Probiotic Supplementation on Intestinal Microbial Composition

**Figure 4:**
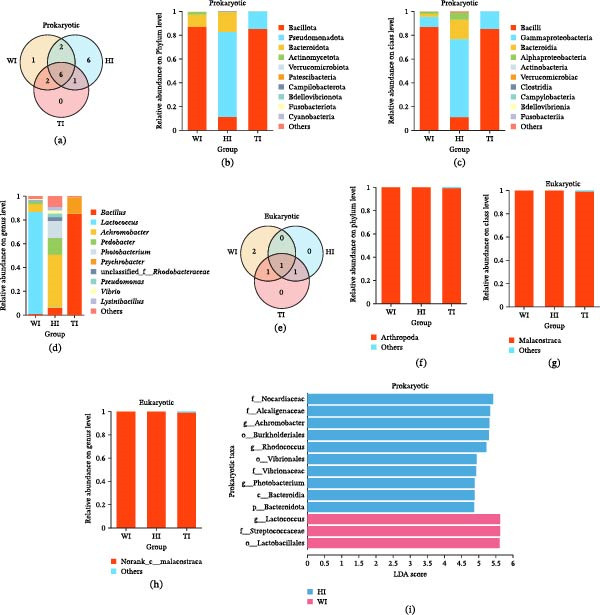
Microbial community composition in shrimp intestinal samples. (a–d) Prokaryotic community composition represented by (a) Venn diagram showing shared and unique prokaryotic phyla across groups. (b–d) Bar plots displaying the relative abundance of prokaryotic taxa at the (b) phylum, (c) class, and (d) genus levels. (e–h) Eukaryotic community composition represented by (e) a Venn diagram of shared/unique eukaryotic phyla and (g–h) bar plots at the (f) phylum (g) class and (h) genus levels. The *x*‐axis labels indicate sample names (WI, HI, TI; group name + intestine), and the *y*‐axis represents relative abundance. Columns in bar plots are color‐coded by group (blue: H, red: W; group T showed no significant discriminative features). LEfSe analysis identifies differentially abundant (i) prokaryotic taxa among groups (Kruskal–Wallis H test, *p* < 0.05).

High‐throughput 16S rRNA gene sequencing revealed distinct, formulation‐dependent differences in intestinal microbial composition (Figure 4a–d). Across all treatments, six core bacterial phyla were observed (Figure 4a), with three major phyla: *Bacillota*, *Pseudomonadota*, and *Bacteroidota*, being dominant (Figure 4b). Marked differences were observed among treatments. W and T were overwhelmingly dominated by *Bacillota* (86.92% and 85.02%, respectively), whereas H exhibited a distinct community structure characterized by enrichment of *Pseudomonadota* (71.38%) and *Bacteroidota* (16.63%). The relative abundance of *Pseudomonadota* in H was ~5‐fold higher than in W and T, while *Bacteroidota* were nearly absent in T. At the class level (Figure 4c), microbial diversity was highest in H (24 classes), followed by W (17 classes) and T (11 classes). *Bacilli* dominated W and T (>85%), whereas H showed a more balanced distribution, with *Gammaproteobacteria* significantly enriched (65.0%; *p* < 0.05).

Genus‐level analysis (Figure 4d) further highlighted treatment‐specific patterns. H exhibited the highest genus richness (134 genera), compared to W (97 genera) and T (26 genera). In contrast, T was strongly dominated by *Bacillus* (85.01%), whereas H showed enrichment of diverse genera, including *Achromobacter*, *Pedobacter*, *Photobacterium*, and *Labrenzia*. *Lactococcus* dominated W (85.87%) but was markedly reduced in H and absent in T. Differential enrichment analysis (Figure 4i) confirmed distinct microbial signatures among treatments, with H showing enrichment of multiple taxa across different taxonomic levels, whereas T showed no significant enrichment. Analysis of 18S rRNA sequences revealed low eukaryotic diversity across all treatments, with no significant differences observed (Figure 4f–h).

### 3.5. Functional Divergence of Intestinal Microbiota Under Single and Mixed Probiotic Treatments

**Figure 5:**
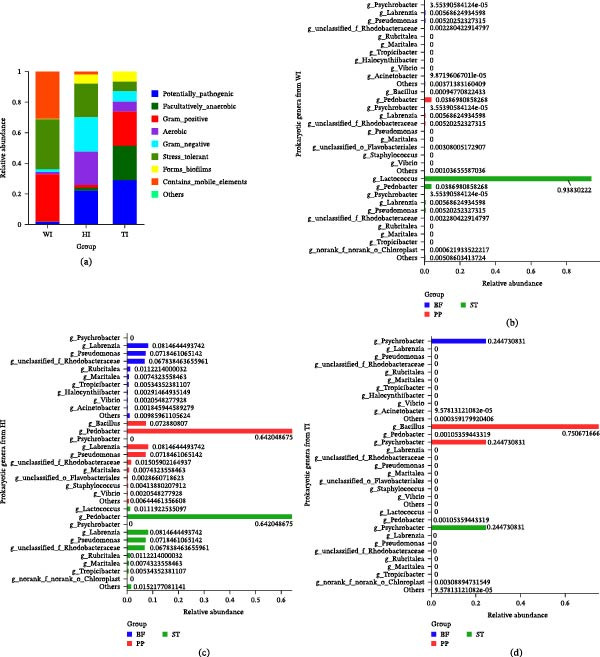
BugBase phenotype analysis of the bacterial community. (a) Bar chart comparing the prevalence of bacterial phenotypes across the three groups (W, H, and T), including stress‐tolerant, potentially pathogenic, biofilm‐forming, Gram‐negative, Gram‐positive, aerobic, anaerobic, facultatively anaerobic, and mobile element‐containing bacteria. (b–d) Detailed relative abundance and taxonomic composition of key functional groups, specifically potentially pathogenic, biofilm‐forming, and stress‐tolerant bacteria, for each intestinal sample group: (b) WI, (c) HI, and (d) TI (sample naming convention: group name + intestine, i.e., WI = W group intestine, etc.). In panels (b–d), the *x*‐axis represents relative abundance, and the *y*‐axis represents prokaryotic taxa. Color gradients correspond to specific bacterial genera, highlighting compositional variations among the groups. BF, biofilm‐forming; PP, potentially pathogenic; ST, stress‐tolerant.

Figure 5 reveals distinct phenotypic differences among treatment groups. T exhibited higher relative abundances of potentially pathogenic, Gram‐positive, and biofilm‐forming bacteria, whereas H showed higher proportions of aerobic and Gram‐negative bacteria (Figure 5a). Facultatively anaerobic and stress‐tolerant phenotypes were relatively higher in probiotic‐treated groups, while bacteria containing mobile elements were more abundant in W.

At the genus level, clear compositional differences were observed across phenotypes (Figure 5b–d). In the control, stress‐tolerant bacteria were dominated by *Lactococcus*, with minor contributions from *Pedobacter* and *Pseudomonas* (Figure 5b). H was dominated by *Pedobacter* across potentially pathogenic and stress‐tolerant categories, with additional contributions from *Bacillus*, Pseudomonas, and *Labrenzia* (Figure 5c). In contrast, T showed strong dominance of *Bacillus* within the potentially pathogenic category and notable contributions from *Psychrobacter* across both potentially pathogenic and biofilm‐forming phenotypes (Figure 5d).

**Figure 6:**
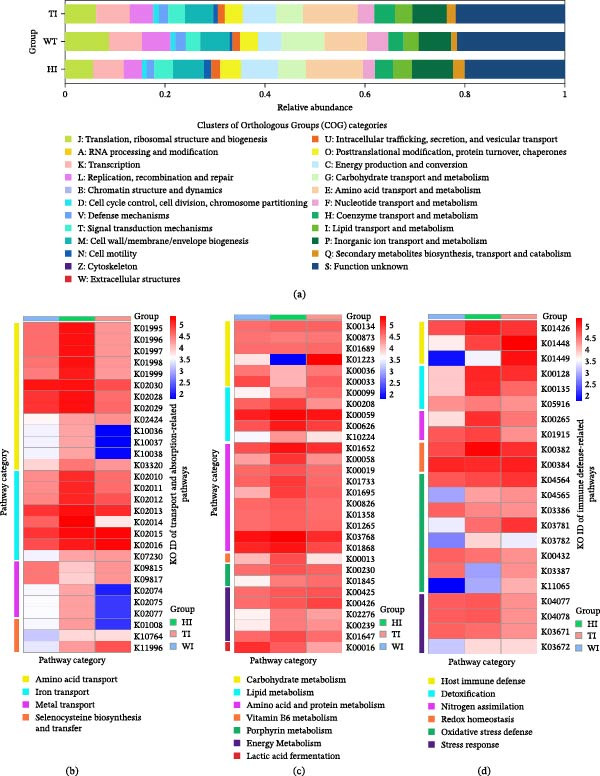
Heatmaps of predicted functional profiles in intestinal bacterial communities across three shrimp groups (W, H, and T) based on PICRUSt2 analysis. (a) Clusters of Orthologous Groups (COG) categories illustrating broad functional differences among groups. The *y*‐axis denotes sample groups (WI, HI, TI; group name + intestine), and the *x*‐axis represents COG categories. (b–d) KEGG pathway‐based heatmaps providing further metabolic and functional insights: (b) KEGG orthologs (KOs) associated with transport and absorption‐related pathways; (c) KOs linked to metabolism‐related pathways; (d) KOs linked to immune defense‐related pathways. In panels (b–d), the *x*‐axis denotes sample groups (WI, HI, TI) and the *y*‐axis represents KO IDs. The color gradient reflects relative functional abundance, as indicated by the legend. Pathway classifications in panels (b–d) are distinguished by color‐coded categories.

The COG‐based functional profile (Figure 6a) revealed distinct functional partitioning between probiotic treatments. The H group (HI) was enriched in biosynthesis and structural/metabolic categories, including amino acid transport and metabolism (E), lipid metabolism (I), cell wall biogenesis (M), motility (N), protein turnover and chaperones (O), inorganic ion transport (P), secondary metabolism (Q), and intracellular trafficking (U). In contrast, T was enriched in energy production and conversion (C), coenzyme metabolism (H), defense mechanisms (V), and signal transduction (T).

Consistent with this, KEGG profiling (Figure 6b–d; Supporting Information 3: Tables S2–S5) showed higher abundances in H of amino acid transport systems (BCAA, polar amino acids, glutamine) and biosynthetic pathways, including amino acid, fatty acid, and nitrogen metabolism (Figure 6b,c; Supporting Information 3: Tables S2–S4). H also exhibited higher iron (Fe) uptake, Zn/Mn transport, and selenocysteine biosynthesis (Figure 6b; Supporting Information 3: Table S2). In contrast, T showed higher enrichment of energy metabolism pathways, including glycolysis and oxidative phosphorylation (Figure 6c; Supporting Information 3: Table S4), alongside increased iron‐related pathways (heme degradation and synthesis) and calcium transport (Figure 6b; Supporting Information 3: Table S2), and elevated immune and oxidative stress defense functions (Figure 6d; Supporting Information 3: Table S5). On the other hand detoxification, nitrogen metabolism and redox homeostasis related pathways was abundant in H than in T and W (Figure 6d; Supporting Information 3: Table S5).

### 3.6. Effects of Probiotic Supplementation on Shrimp Meat Proximate and Micronutrient Composition

Probiotic supplementation significantly affected the proximate composition of shrimp meat (Table 5). H exhibited the highest crude protein content, which was significantly greater than that of the control (*p* < 0.05), while T showed an intermediate value without significant difference from H. Crude lipid content was markedly increased in H, followed by T, with both treatments significantly exceeding the control (*p* < 0.05). A similar trend was observed for moisture content, where H showed the highest value, followed by T, both significantly higher than the control. In contrast, ash content did not differ significantly among treatments (*p* > 0.05).

**Table 5:** The proximate components of meat samples from three groups of shrimp.

Proximate components (%)	WM	HM	TM
Crude protein	11.82 ± 0.01^b^	12.38 ± 0.96^a^	12.04 ± 0.88^a^
Crude lipid/fat	3.09 ± 0.22^b^	4.05 ± 0.56^a^	3.49 ± 0.02^ab^
Ash contents	2.78 ± 2.02^a^	2.92 ± 1.49^a^	2.77 ± 0.86^a^
Moisture contents	73.25 ± 0.04^b^	75.89 ± 1.41^a^	74.57 ± 1.54^a^

*Note:* Data are mean ± SD (*n* = 3 replicate ponds per treatment: WS1–WS3, HS1–HS3, TS1–TS3). Different superscript letters in the same column denote statistically significant differences (*p* < 0.05), while identical letters indicate no significant differences (*p* > 0.05). WM, HM, and TM denote the meat samples from groups W, H, and T, respectively.

Significant variations in mineral composition were observed among treatments (Table 6). H exhibited markedly higher concentrations of several minerals, particularly selenium (Se), iron (Fe), and phosphorus (P), compared to both W and T (*p* < 0.05). T showed intermediate levels of these micronutrients, generally higher than W but significantly lower than H. In contrast, calcium (Ca) was significantly reduced (~50%) in H, decreasing from 162.44 ± 31.8 µg/g in W to 80.91 ± 8.81 µg/g in H. Other minerals, such as Mg, showed moderate increases, whereas Cu and Zn remained relatively stable across treatments, with no statistically significant differences (*p* > 0.05).

**Table 6:** The mineral composition of meat samples from three groups of shrimp.

Minerals (µg/g)	WM	HM	TM
Na	287.57 ± 9.44^a^	482.08 ± 92.16^a^	493.69 ± 140.23^a^
K(g/L) ^∗^	4.39 ± 1.40^a^	5.06 ± 1.37^a^	6.35 ± 0.33^a^
Mg	60.8 ± 9.60^a^	76.63 ± 12.07^a^	64.45 ± 9.86^a^
Ca	162.44 ± 31.8^a^	80.91 ± 8.81^b^	110.8 ± 45.56^ab^
Cu	1.99 ± 0.69^a^	1.74 ± 0.49^a^	1.82 ± 0.10^a^
Se	336.86 ± 18.10^b^	603.38 ± 134.38^a^	362.13 ± 45.71^b^
Fe	16.52 ± 2.99^c^	30.6 ± 8.67^a^	22.26 ± 4.00^b^
P	447.51 ± 35.26^b^	632.04 ± 65.73^a^	504.6 ± 46.73^b^
Zn	3.19 ± 0.96^b^	3.93 ± 1.05^a^	3.59 ± 0.30^b^

*Note:* Data are mean ± SD (*n* = 3 replicate ponds per treatment: WS1–WS3, HS1–HS3, TS1–TS3). Different superscript letters in the same column denote statistically significant differences (*p* < 0.05), while identical letters indicate no significant differences (*p* > 0.05). WM, HM, and TM denote the meat samples from groups W, H, and T, respectively.  ^∗^ indicates the higher concentration of K.

### 3.7. Color Parameters of Shrimp Meat

Significant treatment effects were observed for both raw and cooked shrimp meat color (Table 7). In raw meat, H and W exhibited higher lightness (*L*  ^∗^) than T, while redness (*a*  ^∗^) remained close to zero across groups, with W showing a slightly less negative value. Raw yellowness (*b*  ^∗^) was significantly reduced in the probiotic‑treated groups (T and H) compared with W, indicating lower formation of oxidation‐derived pigments. Cooking increased all color parameters, with W showing the highest cooked *L*  ^∗^ and *b*  ^∗^, whereas T and H consistently displayed lower *b*  ^∗^ values. Both HM and TM exhibited significantly lower total color change (Δ*E* ≈38.3–38.7; *p*  < 0.05) compared to WM (39.44).

**Table 7:** Color parameters of shrimp meat across all groups (W, H, T).

Group	Raw meat			Boiled meat			Total color change (Δ*E*)
	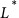	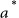	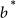	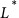	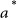	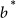	
WM	40.33 ± 0.03^a^	−0.41 ± 0.01^b^	1.78 ± 0.29^a^	66.36 ± 2.39^b^	15.45 ± 0.27^a^	26.81 ± 1.86^a^	39.44 ± 1.45^b^
HM	40.18 ± 0.08^a^	−0.63 ± 0.01^a^	1.35 ± 0.81^b^	64.95 ± 2.04^a^	15.43 ± 0.10^a^	25.78 *b* ± 1.28^b^	38.32 ± 1.01^a^
TM	39.19 ± 0.03^b^	−0.63 ± 0.01^a^	1.38 ± 0.42^b^	65.68 ± 1.37^a^	15.42 ± 0.05^a^	25.68 ± 1.08^b^	38.70 ± 1.03^a^

*Note:* Data are mean ± SD (*n* = 3 replicate ponds per treatment: WS1–WS3, HS1–HS3, TS1–TS3). Different superscript letters in the same column denote statistically significant differences (*p* < 0.05), while identical letters indicate no significant differences (*p* > 0.05). Abbreviations: WM, HM, and TM denote the meat samples from groups W, H, and T, respectively.

### 3.8. Proteomic Modulation of Shrimp Meat Quality

**Figure 7:**
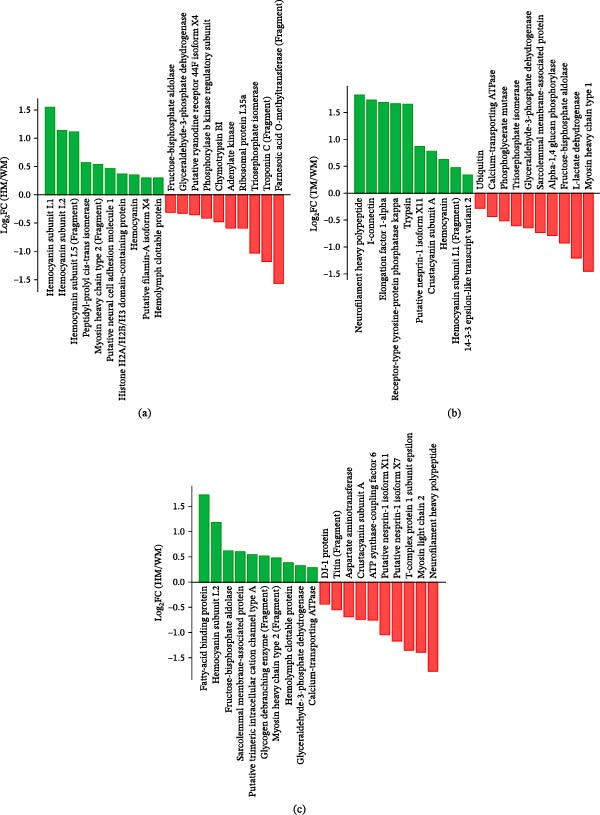
Presents comparative analyses of differentially expressed proteins (DEPs) among shrimp groups: (a) HM vs. WM, (b) TM vs. WM, and (c) HM vs. TM. Each panel displays upregulated (green) and downregulated (red) DEPs as distinct colored bars, highlighting significant expression differences between groups. Sample groups are denoted as WM, HM, and TM (group name + meat).

Proteomic profiling using 4D‐DIA identified 6979 peptides corresponding to 749 proteins across all experimental groups (Supporting Information 3: Table S9), revealing distinct probiotic‐mediated regulatory patterns. Compared with the control (W), the single‐strain probiotic (H) induced 176 DEPs (107↑/69↓), characterized by significant upregulation of hemocyanin subunits and myosin heavy chain type 2 (Figure 7a; Supporting Information 3: Table S10). In contrast, the mixed‐strain probiotic (T) exhibited broader protein suppression (220 DEPs; 96↑/124↓) while selectively enhancing structural proteins such as neurofilament heavy polypeptide and I‐connectin (Figure 7b). Direct comparison between probiotic treatments (H vs. T) identified 183 DEPs (110↑/73↓), confirming distinct regulatory strategies (Figure 7c; Supporting Information 3: Table S10–S17).

**Figure 8:**
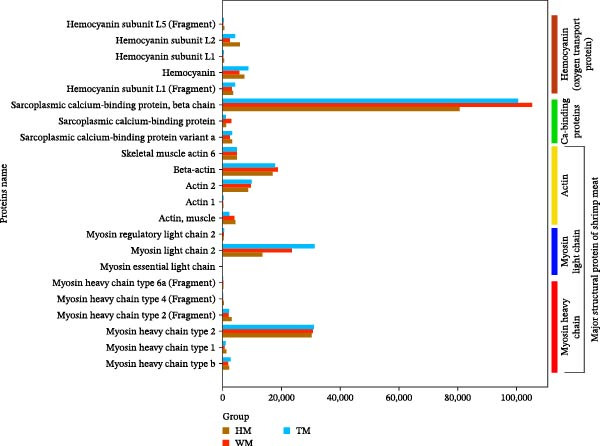
Comparative expression profiles of structural and oxygen‐transport differentially expressed proteins (DEPs) in shrimp meat tissue across three experimental groups (W, H, and T). Protein categories include structural proteins (actin and myosin), oxygen‐transport proteins (hemocyanin subunits), and sarcoplasmic calcium‐binding protein. Sample groups are denoted as WM, HM, and TM (group name + meat).

Hemocyanin isoforms were differentially regulated (Figure 8; Supporting Information 3: Table S11), with isoform Q26180 showing greater induction in T (19.69%) than H, whereas subunit L1, L2, and L5 was markedly elevated in H (77.25%, 38.44%, and 66.53%; H vs. T). Additionally, Myosin heavy chain type 2 (MHC2) was significantly higher in H (28.46%–31.76%) when accompanied by reduced abundance of sarcoplasmic calcium‐binding protein, SCP‐β chain (T:19.85%; W:23.44%) and myosin light chain 2 (MLC2) was strongly downregulated in H than (T:56.5%; W:42.06%).

**Figure 9:**
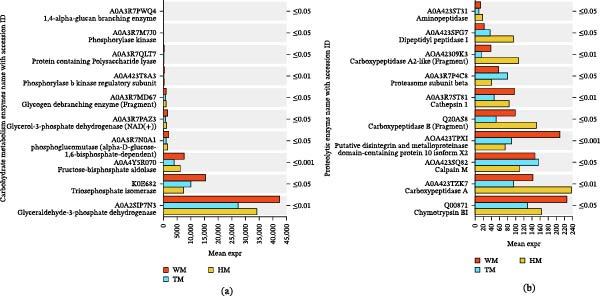
Differential expression patterns of meat quality‐associated proteins across experimental groups W, H, and T. Bar charts showing significant variations in differentially expressed proteins (DEPs) among the W, H, and T groups for two functional categories: (a) Glycolytic enzymes; (b) Proteolytic enzymes. The horizontal axis represents the mean relative protein expression across groups (WM, HM, TM; group name + meat sample), with different colored bars representing distinct groups. The vertical axis indicates protein designations. Red, blue, and orange markers correspond to groups W, H, and T, respectively. The *p*‐value is indicated on the far right, where 0.01 < *p* ≤ 0.05,  ^∗^ 0.001< *p* ≤ 0.01,  ^∗∗∗^ *p* ≤ 0.001.

Both probiotic treatments consistently downregulated key glycolytic enzymes, including triosephosphate isomerase (TPI), glyceraldehyde‐3‐phosphate dehydrogenase (GAPDH), fructose‐bisphosphate aldolase (FBA), phosphoglycerate mutase, and L‐lactate dehydrogenase (LDH) (Figure 9a; Supporting Information 3: Table S12), indicating reduced glycolytic activity.

Proteolytic (flavor‐related) enzyme profiles revealed treatment‐specific patterns (Figure 9b; Supporting Information 3: Table S13). HM exhibited strong downregulation of chymotrypsin BII, calpain M, and cathepsin L, indicating reduced postmortem proteolysis. Carboxypeptidase A1 and dipeptidyl peptidase 1 were elevated in HM, suggesting selective peptide trimming rather than broad protein degradation.

**Figure 10:**
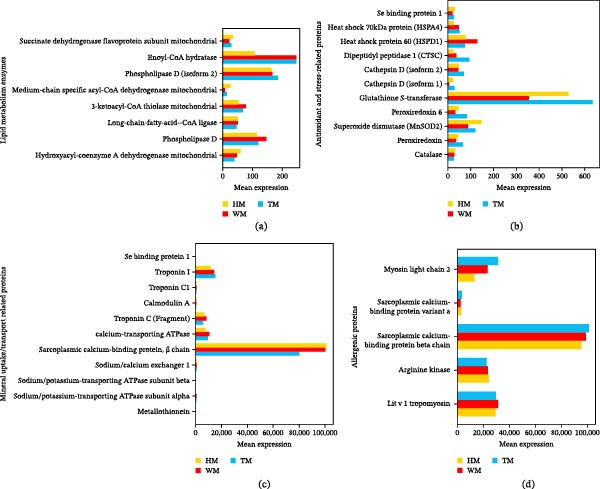
Differential expression patterns of meat quality‐associated proteins across experimental groups W, H, and T. Bar charts showing significant variations in differentially expressed proteins (DEPs) among the W, H, and T groups for four functional categories: (a) Lipid metabolism and oxidation‐related proteins; (b) Antioxidant‐related proteins; (c) Mineral‐binding proteins; (d) Allergenic (allergy‐related) proteins. The *x*‐axis represents the mean relative protein expression, and the *y*‐axis indicates protein designations. Sample groups are denoted as WM, HM, and TM (group name + meat sample). Different colored bars represent distinct groups, with red, blue, and yellow markers corresponding to groups W, T, and H, respectively.

The analysis of lipid metabolism and oxidative stability revealed distinct group‐specific patterns (Figure 10a; Supporting Information 3: Table S14). Both probiotic treatments downregulated lipid‐degrading enzymes, including glycerol‐3‐phosphate dehydrogenase and phospholipase D. Additionally, mitochondrial 3‐ketoacyl‐CoA thiolase abundance decreased by 28.6% in H and 18.6% in T, indicating reduced terminal β‐oxidation. Concurrently, H maintained higher levels of primary antioxidants (catalase and MnSOD2) via the peroxisome pathway (Figure 10b; Supporting Information 3: Table S15). In contrast, W and T shifted toward secondary antioxidant systems, characterized by elevated peroxiredoxin, peroxiredoxin 6, glutathione S‐transferase (GST), along with increased heat shock proteins (HSPs) and cathepsin D.

The analysis of mineral absorption proteins revealed distinct tissue‐specific patterns (Figure 10c; Supporting Information 3: Table S16). Sarcoplasmic calcium‐binding proteins were most abundant in T and W samples (~100,000), reflecting active muscle contraction. Similarly, calcium‐ATPase and calmodulin were higher in W, suggesting enhanced calcium sequestration in shrimp meat. Sodium/potassium ATPase and sodium/calcium exchanger 1 (NCX1) were highest in H, indicating active ion transport. Notably, metallothionein was detected only in *H* (8.76) and *T* (5.87) but was completely absent in W. Regarding allergenicity, major allergens (tropomyosin and arginine kinase) showed no significant changes, whereas minor allergens, including sarcoplasmic calcium‐binding protein, and myosin light chain 2 were significantly modulated (*p* < 0.05; Figure 9d; Supporting Information 3: Table S17). Overall, both probiotic treatments improved meat quality compared to the control, with H showing superior advantages across multiple parameters, namely structural integrity, color stability, oxidative stability, nutritional minerals, and allergen reduction as summarized in Table 8.

**Table 8:** Comparative efficacy of single‐strain (H) versus mixed‐strain (T) probiotics on shrimp meat quality attributes relative to untreated control (W).

Quality attribute	H (single strain)	T (mixed strain)	Mechanistic basis
Structural integrity	+++	++	MHC2↑, proteolytic enzymes↓
Color stability	+++	++	Hemocyanin↑, antioxidant balance↑
Oxidative stability	+++	+	Thiolase↓, primary antioxidants↑
Nutritional minerals	+++	++	Metallothionein↑ (absent in W), Se↑
Glycolytic suppression	++	+++	GAPDH↓, LDH↓, TPI↓
Allergen reduction	++	+	SCP‐β↓, MLC2↓

*Note:* +++ = strongest improvement; ++ = moderate improvement; + = slight improvement. Mechanistic basis derived from 4D‐DIA proteomic profiling (Supporting Tables S11–S17).

Abbreviations: LDH, lactate dehydrogenase; MHC2, myosin heavy chain type 2; MLC2, myosin light chain 2; SCP‐β, sarcoplasmic calcium‐binding protein beta chain; Se, selenium; TPI, triosephosphate isomerase.

## 4. Discussion

The present study demonstrates that probiotic supplementation markedly enhances growth performance, feed efficiency, survival, intestinal functionality, and meat quality in *L. vannamei* by reshaping the gut microbial ecosystem. Notably, these benefits were strongly formulation‑dependent: the single‑strain probiotic *L. salivarius* GZPH2 (H) consistently outperformed both the mixed‐strain probiotic (T) and the control (W). These findings indicate that probiotic efficacy is not simply determined by microbial abundance or strain number, but rather by the ability to establish a balanced, functionally integrated gut microbiome.

### 4.1. Probiotics Improve Growth Performance Through Microbiota‐Mediated Nutrient Utilization

Both probiotic treatments significantly improved growth performance and FCR (Table 1), consistent with previous studies demonstrating probiotic benefits in shrimp aquaculture [24, 25]. However, the superior performance observed in the H group suggests that microbial functional balance and diversity are more critical than dominance‐driven microbial proliferation [26, 27]. This conclusion is supported by microbial community analysis (Figure 4a–d), which showed that H maintained significantly higher diversity and evenness (Tables 3 and 4), while T was dominated by *Bacillus* (~85%) and W by *Lactococcus*. High microbial diversity is known to enhance functional redundancy, metabolic flexibility, and nutrient conversion efficiency, thereby improving host growth [28]. In contrast, dominance‐driven systems, such as in T, reduce ecological complexity and limit metabolic cooperation, resulting in comparatively lower performance despite strong colonization ability. Survival rates followed a similar pattern (H > T > W; Table 1), further indicating that a stable and balanced microbiome enhances host resilience more effectively than a competitive, dominance‐driven system.

### 4.2. Probiotic‑Driven Restructuring of Intestinal Microbiota and Mechanistic Differences Between Single‑ and Mixed‑Strain Probiotics

Probiotic supplementation induced clear, treatment‑specific restructuring of the intestinal microbiota. The single‑strain probiotic (H) exhibited significantly higher α‑diversity than the mixed‑strain (T) (Tables 3 and 4), reflecting a more balanced microbial ecosystem without dominance by a single taxon. H displayed the highest richness (134 genera) and evenness (Table 3) and was enriched with metabolically versatile taxa, including *Achromobacter*, *Pedobacter*, *Photobacterium*, and *Labrenzia* (Figures 4 and 5), many of which showed positive correlations with shrimp growth and meat yield (Supporting Information 1: Figure 7a). These taxa contribute to nutrient cycling, mineral solubilization, and metabolic cooperation, thereby supporting host performance [29, 30]. KEGG functional profiling further revealed that H was enriched in diverse nutrient‑acquisition and biosynthetic pathways, including amino acid transport systems (branched‑chain, glutamine, and polar amino acids), protein‑biosynthesis pathways, fatty‑acid biosynthesis, and iron and trace‑mineral transport modules (Figure 6b–d; Supporting Information 3: Tables S2–S4). These functions support efficient nutrient digestion, absorption, and assimilation, aligning with improved growth and feed utilization reported in aquatic species [6, 31]. Enhanced amino‑acid and nitrogen metabolism promotes protein turnover and muscle accretion [32], while fatty‑acid biosynthesis contributes to energy utilization and lipid deposition [33]. Enrichment of detoxification and redox‑homeostasis pathways, including aldehyde dehydrogenase, glutamine synthetase, and thioredoxin systems (Supporting Information 3: Table S5), suggests reduced oxidative and metabolic stress. Strengthened antioxidant capacity is essential for maintaining intestinal integrity under intensive aquaculture conditions [34, 35]. These functional pathways help to balance the gut environment and reduce competitive exclusion [26]. These features promote niche partitioning and functional complementarity, enabling multiple microbial taxa to coexist and collectively contribute to host nutrition and metabolism. This is consistent with previous findings that higher microbial diversity enhances metabolic versatility and host growth [27, 36].

In contrast, the reduction in microbial diversity in the mixed‑strain probiotic group (T) is primarily attributable to *Bacillus*‑driven competitive exclusion, supported by functional and genomic evidence. T exhibited strong *Bacillus* dominance (Figures 4d and 5a), accompanied by enrichment of colonization and competitive traits, including adhesion‑related genes (e.g., type IV pili) and antimicrobial‑ or toxin‑associated genes such as hemolysins, enterotoxins, and thiol‑activated cytolysin (Supporting Information 3: Tables S6 and S7). These factors suppress competing commensals [37, 38]. KEGG analysis also indicated elevated antimicrobial and stress‑response functions (Figure 6d; Supporting Information 3: Table S5), consistent with a defense‑oriented microbiome. Such antimicrobial pressure reduces community evenness and eliminates low‑abundance taxa, a pattern widely reported for *Bacillus*‑based probiotics in shrimp [39, 40]. Previous studies confirm that *Bacillus* spp. exert antagonistic activity through N‑acetylmuramoyl‑L‑alanine amidase (Supporting Information 3: Table S5), a bacterial cell‑wall hydrolase that drives niche exclusion and diversity loss [41, 42]. BugBase further classified “potentially pathogenic” bacteria as more abundant in T, including *Bacillus* (Figure 5c), likely due to toxin‑related and antimicrobial genes; however, in aquaculture contexts, these functions primarily mediate microbial competition rather than host pathogenicity, consistent with the absence of disease symptoms. Thus, the primary driver of reduced diversity in T is antimicrobial‑mediated competitive exclusion by dominant *Bacillus* populations.

Dominance‑driven communities in T and W also exhibited distinct metabolic partitioning. Carbohydrate metabolism was channeled into specialized, less integrated pathways (Figure 6c; Supporting Information 3: Table S4). In T, *Bacillus* dominance favored extracellular polysaccharide depolymerization and intensified central carbon metabolism, reflected by higher abundances of glyceraldehyde‑3‑phosphate dehydrogenase, glucose‑6‑phosphate dehydrogenase, and enolase—consistent with the metabolic profile of *Bacillus* spp. [43]; (Acharya and Subedi 2025). Conversely, *Lactococcus* dominance in W redirected carbohydrate utilization toward fermentative glycolysis, indicated by elevated L‑lactate dehydrogenase (Figure 6c), supporting increased glycolytic flux and lactate production [44]. These patterns demonstrate that dominance‑structured microbiomes prioritize rapid energy turnover and competitive advantage but sacrifice metabolic versatility compared with more functionally balanced communities. Overall, single‑strain probiotic supplementation created a more diverse, metabolically versatile, and functionally cooperative gut microbiome than the mixed‑strain treatment. This enhanced microbial complementarity translated into superior nutrient assimilation, redox stability, and overall host performance.

### 4.3. Intestinal Remodeling Enhances Nutrient Absorption and Meat Quality

Probiotic supplementation induced pronounced intestinal remodeling, resulting in markedly improved nutrient absorption and superior meat quality. The intestinal absorptive area increased substantially in probiotic‑treated shrimp (H > T > W; Table 2), reflecting expansion of epithelial surface architecture that enhances nutrient flux and assimilation. These structural improvements are consistent with recent findings demonstrating that probiotics preserve intestinal morphology, reinforce epithelial integrity, and modulate digestive interfaces in aquatic species [45]. Correspondingly, muscle biochemical composition improved, with elevated crude protein, lipid, and essential mineral (Fe, Se, P) deposition in both H and T groups (Tables 5 and 6), with the strongest effect observed in H. Mucosal protection was further strengthened by increased goblet cell density (T > H > W) and thickening of the muscularis layer, features associated with enhanced mucus secretion, epithelial barrier stability, and regulated host–microbe interactions (Gustafsson and Johansson 2022). Contemporary reviews underscore the central role of goblet cells in immune tolerance, microbial homeostasis, and epithelial defense [46].

Functionally, KEGG pathway enrichment revealed coordinated upregulation of nutrient‑transport and metabolic modules (Figure 6b,c; Supporting Information 3: Tables S2–S4). In the H group, enrichment of Fe^3+^ transport systems, including ABC transporters (K02010–K02016), outer‑membrane receptors, and periplasmic binding proteins, together with elevated ferroxidase activity indicates enhanced iron uptake, oxidation, and intracellular trafficking, consistent with the higher Fe concentration observed in muscle [47, 48]. Similarly, upregulation of selenocysteine biosynthesis pathways, coupled with higher Se levels in H (H > T), suggests more efficient conversion of selenium into functional selenoproteins, supporting antioxidant defense and metabolic regulation [49].

Mineral interaction patterns further revealed mechanistic trade‑offs. Calcium, a well‑established antagonist of iron absorption, displayed an inverse relationship with Fe across treatments (Ca: W > T > H; Fe: H > T > W; Table 5). This pattern aligns with evidence that Ca competes with Fe at the transporter level, reducing Fe uptake when Ca transport is elevated [28, 50]. Thus, the downregulation of Ca‑transport pathways in H likely alleviated competitive inhibition, contributing to enhanced Fe absorption.

Microbiota‑level shifts also supported improved mineral assimilation. Enrichment of mineral‑solubilizing taxa in H such as *Achromobacter*, *Pseudomonas*, and *Labrenzia* (Figures 4d and 5c) likely facilitated mineral mobilization through chelation, siderophore production, and redox transformation [51–53]. Enhanced metabolic capacity in H, evidenced by upregulated branched‑chain amino acid (BCAA) metabolism and fatty‑acid biosynthesis, corresponded with higher protein (12.38%) and lipid (4.05%) deposition, consistent with probiotic‑driven anabolic enhancement [54]. Collectively, these results demonstrate that probiotics, particularly strain H, optimize gut structural–functional coupling and microbiota‑mediated nutrient dynamics, culminating in markedly improved shrimp meat quality.

### 4.4. Proteomic Modulation of Shrimp Meat Quality by Probiotics

Proteomic profiling revealed that probiotic supplementation, particularly the single‑strain *Lactobacillus salivarius* GZPH2, modulates multiple biochemical pathways that collectively enhance shrimp meat quality. A total of 749 proteins were identified (Supporting Information 3: Table S9), providing a mechanistic framework linking probiotic‑driven host–microbe interactions to improvements in structural integrity, metabolic efficiency, oxidative stability, and nutritional value.

The most prominent improvement was the preservation of myofibrillar proteins, with myosin heavy chain type 2 (MHC2) significantly elevated in the H group (Figure 8; Supporting Information 3: Table S11). Enhanced MHC2 abundance indicates superior maintenance of the contractile apparatus, consistent with previous findings that MHC stability is a key determinant of texture, water‑holding capacity, and cooking yield [55, 56]. Protease profiles further supported this structural protection: the H group strongly downregulated calpain M, cathepsin L, and chymotrypsin BII (Figure 9b; Supporting Information 3: Table S13), reducing postmortem proteolysis that typically accelerates softening and drip loss [57–59]. Correspondingly, moisture retention improved in both probiotic groups (H: +2.79%; T: +1.63%; Table 5). In contrast, T exhibited weaker protease suppression. Notably, H upregulated carboxypeptidase A1 and dipeptidyl peptidase 1, suggesting a shift toward selective peptide trimming rather than broad degradation, potentially enhancing flavor precursor formation without compromising texture (Toldrá and Flores 1998).

Color stability and oxidative resistance were strongly associated with hemocyanin, which emerged as a central mechanistic node. The H group markedly elevated hemocyanin subunits L1, L2, and L5, whereas T preferentially induced isoform Q26180 (Figure 8; Supporting Information 3: Table S10). Hemocyanin contributes to the characteristic blue‑green hue of crustacean tissues and exhibits phenoloxidase‑like antioxidant activity [60, 61]. Its upregulation in both probiotic groups (H > T > W) corresponds with the superior color stability observed (Table 7), likely through enhanced oxygen binding and reduced pigment oxidation.

Probiotics also modulated postmortem energy metabolism. Glycolytic enzymes, including GAPDH, LDH, TPI, FBA, and phosphoglycerate mutase, were consistently downregulated in probiotic‑treated shrimp (Figure 9a; Supporting Information 3: Table S12), indicating reduced glycolytic flux. This metabolic shift was reflected in higher ultimate pH values (T: 6.10 > H: 6.07 > W: 5.91), consistent with slower lactate accumulation and attenuated postmortem acidification. Reduced glycolysis preserves protein solubility and minimizes drip loss, thereby improving juiciness and texture [55, 62].

Lipid metabolism and oxidative stability were also significantly influenced by probiotics. Downregulation of lipid‑degrading enzymes, glycerol‑3‑phosphate dehydrogenase, phospholipase D, and mitochondrial 3‑ketoacyl‑CoA thiolase indicated reduced terminal β‑oxidation (Figure 10a; Supporting Information 3: Table S14). Because β‑oxidation generates reactive oxygen species (ROS) during mitochondrial decline, limiting this pathway reduces oxidative stress and rancidity [63]. Reduced lipase activity has similarly been shown to improve oxidative stability in muscle foods [64].

Mineral metabolism further contributed to improved nutritional quality. Ion transport proteins displayed treatment‑specific patterns (Figure 10c; Supporting Information 3: Table S16), with Na^+^/K^+^‑ATPase and NCX1 highest in H, suggesting enhanced ion homeostasis and potentially reduced drip loss. The mineral‑binding protein metallothionein was detected only in probiotic groups (H: 8.76 > T: 5.87), and absent in W. Metallothionein binds Fe, Zn, Cu, and Se [65], providing a mechanistic explanation for the elevated selenium and iron concentrations in H‑treated shrimp (Table 6). This aligns with evidence that mineral enrichment improves the nutritional value of aquatic muscle (Lall and Kaushik 2021). The observed presence of Se and metallothionein, coupled with significantly reduced HSP70 expression (Figure 10b; Supporting Information 3: Table S15), indicates that the probiotic‐supplemented group maintained cellular homeostasis and was not subjected to acute or chronic oxidative or thermal stress [66]. This finding is consistent with improved meat quality and enhanced stress resilience.

The elevated Zn/Cu ratio in probiotic‑treated shrimp (H: 2.27 > T: 1.97 > W: 1.60) corresponds with established indicators of oxidative stability and freshness [3]. Increased metallothionein expression likely stabilizes Zn^2+^ and Cu^+^ ions, mitigating oxidative damage (Figure 10b; Supporting Information 3: Table S16) [67]. Differential enhancement of GST and crustacyanin (T > H > W; Figure 10b; Supporting Information 1: Figure S6) further reinforced antioxidant defense and pigment stabilization [68]. Given that protein oxidation is a well‑established marker of meat spoilage [69, 70], these biomarkers complement conventional indicators such as pH decline and color unstability which was more in control group (Table 7; Supporting Information 3: Table S8).

Finally, probiotic supplementation modulated allergenicity without affecting major allergens. Tropomyosin and arginine kinase were not significantly different (*p*  > 0.05) across treatments (Figure 10d; Supporting Information 3: Table S17), indicating that probiotics do not alter primary allergenic risk. However, minor allergens, including SCP‑β and MLC2, were significantly reduced in H, suggesting a modest decrease in allergenic potential without compromising safety (Ayuso et al. 2009; [71, 72].

Overall, probiotic supplementation, especially *L. salivarius* GZPH2, reprograms key structural, metabolic, and antioxidant pathways to produce shrimp meat with superior texture, stability, and nutritional value. These coordinated proteomic shifts highlight probiotics as an effective strategy for enhancing meat quality without altering major allergenic risks.

## 5. Conclusion

This study demonstrates that dietary supplementation with the single‐strain probiotic *L. salivarius* GZPH2 provides a consistent advantage over a mixed‐strain probiotic for *L. vannamei* cultured under tropical conditions. Single‐strain supplementation enhanced growth performance and meat yield, underpinned by improved intestinal morphology, increased nutrient assimilation, and a shift toward a more diverse and metabolically active gut microbiota. In contrast, the mixed‐strain probiotic was less effective due to Bacillus‐driven competitive exclusion and reduced microbial diversity, underscoring that probiotic efficacy depends on functional balance rather than strain abundance alone. Collectively, these findings establish *L. salivarius* GZPH2 as a promising feed additive for targeted probiotic strategies in tropical shrimp aquaculture systems.

## Author Contributions

Conceptualization and supervision: Junpeng Cai. Methodology and investigation: Farhana Najnine, Xinbo Guo, and Junpeng Cai. Formal analysis and writing – original draft: Farhana Najnine. Writing – review & editing: Farhana Najnine and Junpeng Cai.

## Funding

This study was supported by the Science and Technology Department of Guangdong Province of China (2016A020222002) and ProBioti Biotech Company Limited (Guangzhou).

## Disclosure

All authors have read and agreed to the published version of the manuscript.

## Ethics Statement

All animal cultivation procedures followed National Animal Care Guidelines and were approved by the Ethical Committee of the South China University of Technology and Guangdong Province, China. The methods were based on established patents (ZL201410752144.2 and ZL202310736625.3), ensuring no novel interventions that could cause animal harm or distress were used.

## Conflicts of Interest

The authors declare no conflicts of interest.

## Supporting Information

Additional supporting information can be found online in the Supporting Information section.

## Supporting information


**Supporting Information 1** Figure S1: The monthly average maximum and minimum temperature and precipitation (mm) of Haikou City in 2023 (Source: https://www.msn.cn/zh-cn/weather/forecast/). Figure S2. Experimental pond layout and treatment allocation. The study followed a completely randomized design consisting of three dietary treatments, each with three replicate ponds (*n* = 9 ponds total). Nine uniform rectangular earthen ponds (60 m^2^ per pond) were arranged in three parallel rows to minimize cross‐contamination. Each pond was lined with HDPE geomembrane and equipped with an independent aerator and water pump. The pond profile included a dike height of 0.8–1.2 m, a bottom slope of 0.4 m, and a maintained water depth of 0.6–0.9 m (panel B). Treatments included: Control (W): Basal diet only (W1, W2, W3), Commercial probiotic (T): Basal diet + EM mixed‐strain probiotic (T1, T2, T3) and GZPH2 (H): Basal diet + Lactobacillus salivarius GZPH2 (H1, H2, H3). Figure S3. Temporal changes in water nitrogenous metabolites before and after a 30% water exchange during the culture period. Pre‐change (immediately before 30% water exchange: (a) TAN, (b) NO_2_⁻–N, and (c) NO_3_⁻–N. Post‐change (immediately after exchange): (d) TAN, (e) NO_2_⁻–N, and (f) NO_3_⁻–N. Lines indicate the control (W), commercial probiotic (H), and Lactobacillus salivarius GZPH2 (T) groups. Nitrogen concentrations increased over time in all treatments; W showed the highest levels, H intermediate levels, and T the lowest. Water exchange reduced concentrations in all groups, while the relative pattern (W > H > T) remained consistent. Figure S4. Effect of 30% Water exchange on pH dynamics in three aquaculture treatment systems: (a) Pre‐exchange pH: shows the progressive alkalinization during system operation, with all treatments starting at pH 7.5 and diverging to treatment‐specific peaks (W: 8.6, H: 8.35, T: 8.15) due to metabolic accumulation. (b) Post‐exchange pH: demonstrates the moderating effect of water exchange, showing reduced pH extremes (W: 8.25, H: 8.05, T: 7.9) and smoother trajectories while maintaining the consistent treatment hierarchy (W > H > T). Figure S5. Correlation heatmaps investigating the relationships between shrimp biology and microbiome features. The analyses explore correlations between: (a) Intestinal/hindgut morphology and microbiota composition; (b) Microbiota and meat composition (minerals and proximate components); (c) Histology and meat composition. Heatmaps employ a red‐blue color gradient to represent the strength and direction (positive or negative) of Pearson correlation coefficients (r), categorized as follows: very strong (∣*r*∣>0.75∣*r*∣>0.75) to weak (∣*r*∣<0.25∣*r*∣<0.25). Figure S6. Differential expression patterns of meat quality‐associated proteins across experimental groups W, H, and T. Bar charts showing significant variations in differentially expressed proteins (DEPs) among the W, H, and T groups for shrimp meat color related proteins. The horizontal axis represents the mean relative protein expression across groups (WM, HM, TM; group name + meat sample), with different colored bars representing distinct groups. The vertical axis indicates protein designations. Red, blue, and orange markers correspond to groups W, H, and T, respectively. The p‐value is indicated on the far right, where 0.01 < *p* ≤ 0.05,  ^∗^0.001< *p* ≤ 0.01,  ^∗∗∗^ *p* ≤ 0.001.

**Supporting Information 2** Note S1: Water Quality Testing Method. Note S2. Probiotic sources, preparation and application of commercial EM probiotic. and Lactobacillus salivarius strain GZPH2 for shrimp feed supplementation. Note S3. Growth performance and yield parameters related formulas. Note S4. pH Measurement of Shrimp Meat. Note S5. Meat Color Measurement (Raw and Boiled Shrimp).

**Supporting Information 3** Table S1: The nutritional composition of feed (In supporting tables file: page 1). Table S2. KEGG‐based profiling of absorption‐related pathways in intestinal microbiota across treatments W, H, and T (In supporting tables file: start from page 1 continue to page 4). Table S3. KEGG‐based annotation of mineral (ion) metabolism and transport in intestinal microbiota across treatments W, H, and T (In supporting tables file: start from page 4 continue to page 5). Table S4. KEGG‐based annotation of metabolism‐related enzymes in intestinal microbiota across treatments W, H, and T (In supporting tables file: start from page 6 continue to page 9). Table S5. KEGG‐based annotation of immunity‐related enzymes in intestinal microbiota across treatments W, H, and T (In supporting tables file: start from page 9 continue to page 12). Table S6. KEGG‐based annotation of toxin‐related (virulence‐associated) genes in intestinal microbiota across treatments W, H, and T (In supporting tables file: start from page 12 continue to page 13). Table S7. KEGG‐based annotation of adhesion, colonization, and secretion system‐related genes in intestinal microbiota across treatments W, H, and T (In supporting tables file: start from page 13 continue to page 14). Table S8. Post‐mortem pH changes of shrimp meat (0–24 h) across treatments W, H, and T (In supporting tables file: page 14). Table S9. Full protein functional annotation table (In supporting tables file: page 15). Table S10. Statistical table of the number of differential proteins in shrimp meat across treatments W, H, and T (In supporting tables file: page 15). Table S11. Protein abundance of major structural, calcium‑binding, and oxygen‑transport proteins in shrimp meat across group W, H, and T (In supporting tables file: start from page 15 continue to page 17). Table S12. Differential expression of carbohydrate metabolism enzymes and pathways in shrimp muscle across group W, H, and T (In supporting tables file: start from page 17 continue to page 18). Table S13. Differential expression of proteolytic enzyms in shrimp meat across group W, H, and T (In supporting tables file: start from page 18 continue to page 19). Table S14. Differential expression of lipid metabolism enzymes in shrimp meat across group W, H, and T (In supporting tables file: start from page 19 continue to page 20). Table S15. Differential expression of antioxidant and stress‐related proteins in shrimp meat across group W, H, and T (In supporting tables file: start from page 20 continue to page 21). Table S16. Differential expression of mineral absorption/uptake/transport related proteins in shrimp meat across group W, H, and T (In supporting tables file: start from page 21 continue to page 22). Table S17. Differential expression of major and minor allergenic protein in shrimp meat across group W, H, and T (In supporting tables file: page 22).

## Data Availability

Data will be made available upon request.
