# Supplementary material for: Lactobacillus salivarius Probiotic Supplementation Modulates Gut Function, Improves Growth, and Meat Quality in Tropical Whiteleg Shrimp
Source: Aquac Nutr. 2026 May 27;2026:6285997. doi: 10.1155/anu/6285997 (PMC13213333; doi:10.1155/anu/6285997)
Supplement: Supplementary file 1 — Supporting Information 1 Figure S1: The monthly average maximum and minimum temperature and precipitation (mm) of Haikou City in 2023 (Source: https://www.msn.cn/zh-cn/weather/forecast/). Figure S2. Experimental pond layout and treatment allocation. The study followed a completely randomized design consisting of three dietary treatments, each with three replicate ponds (n = 9 ponds total). Nine uniform rectangular earthen ponds (60 m2 per pond) were arranged in three parallel rows to minimize cross‐contamination. Each pond was lined with HDPE geomembrane and equipped with an independent aerator and water pump. The pond profile included a dike height of 0.8–1.2 m, a bottom slope of 0.4 m, and a maintained water depth of 0.6–0.9 m (panel B). Treatments included: Control (W): Basal diet only (W1, W2, W3), Commercial probiotic (T): Basal diet + EM mixed‐strain probiotic (T1, T2, T3) and GZPH2 (H): Basal diet + Lactobacillus salivarius GZPH2 (H1, H2, H3). Figure S3. Temporal changes in water nitrogenous metabolites before and after a 30% water exchange during the culture period. Pre‐change (immediately before 30% water exchange: (a) TAN, (b) NO2⁻–N, and (c) NO3⁻–N. Post‐change (immediately after exchange): (d) TAN, (e) NO2⁻–N, and (f) NO3⁻–N. Lines indicate the control (W), commercial probiotic (H), and Lactobacillus salivarius GZPH2 (T) groups. Nitrogen concentrations increased over time in all treatments; W showed the highest levels, H intermediate levels, and T the lowest. Water exchange reduced concentrations in all groups, while the relative pattern (W > H > T) remained consistent. Figure S4. Effect of 30% Water exchange on pH dynamics in three aquaculture treatment systems: (a) Pre‐exchange pH: shows the progressive alkalinization during system operation, with all treatments starting at pH 7.5 and diverging to treatment‐specific peaks (W: 8.6, H: 8.35, T: 8.15) due to metabolic accumulation. (b) Post‐exchange pH: demonstrates the moderating effect of water exchange [file ANU-2026-6285997-s003.docx]

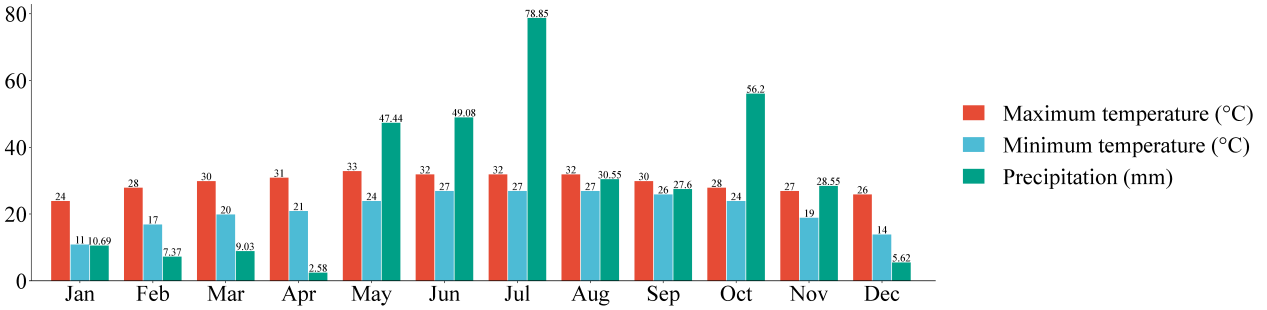


**Supplementary Figure S1.** The monthly average maximum and minimum temperature and precipitation (mm) of Haikou City in 2023 (Source: https://www.msn.cn/zh-cn/weather/forecast/)


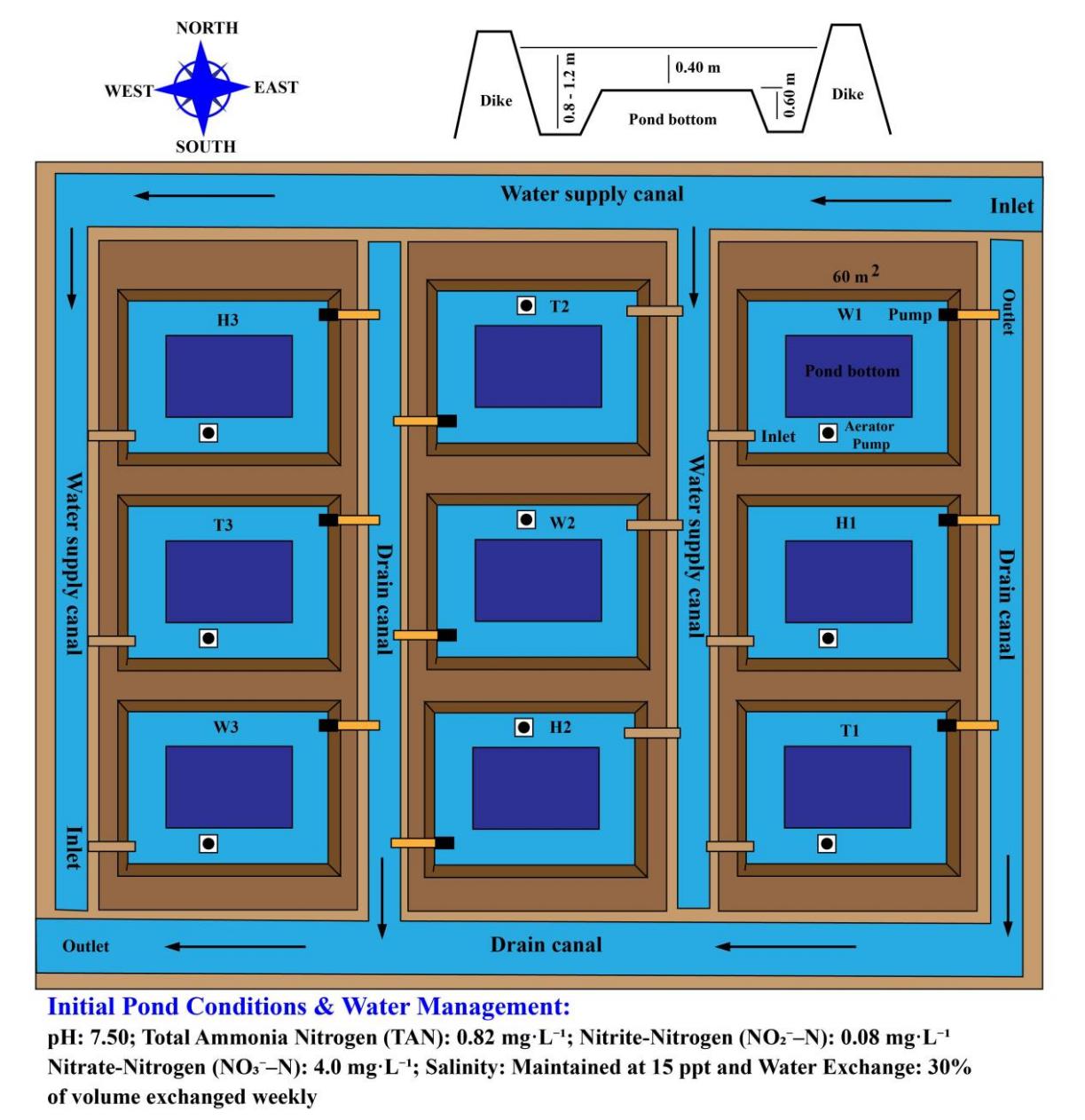


**Supplementary Figure S2.** Experimental pond layout and treatment allocation. The study followed a completely randomized design consisting of three dietary treatments, each with three replicate ponds (n = 9 ponds total). Nine uniform rectangular earthen ponds (60 m² per pond) were arranged in three parallel rows to minimize cross-contamination. Each pond was lined with HDPE geomembrane and equipped with an independent aerator and water pump. The pond profile included a dike height of 0.8–1.2 m, a bottom slope of 0.4 m, and a maintained water depth of 0.6–0.9 m (panel B). Treatments included: Control (W): Basal diet only (W1, W2, W3), Commercial probiotic (T): Basal diet + EM mixed-strain probiotic (T1, T2, T3) and GZPH2 (H): Basal diet + *Lactobacillus salivarius* GZPH2 (H1, H2, H3).

**
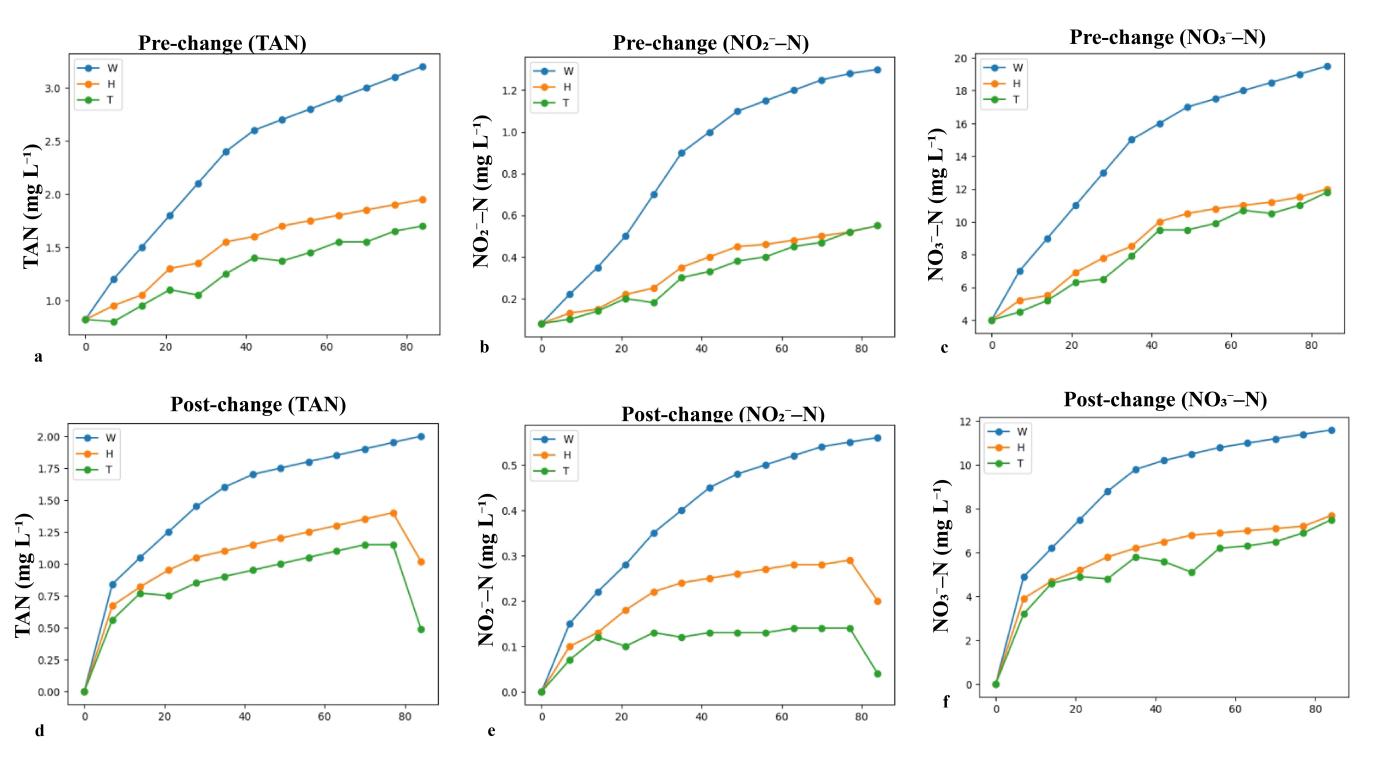
**

**Supplementary Figure S3.** Temporal changes in water nitrogenous metabolites before and after a 30% water exchange during the culture period. Pre-change (immediately before 30% water exchange: (a) TAN, (b) NO₂⁻–N, and (c) NO₃⁻–N. Post-change (immediately after exchange): (d) TAN, (e) NO₂⁻–N, and (f) NO₃⁻–N. Lines indicate the control (W), commercial probiotic (H), and *Lactobacillus salivarius* GZPH2 (T) groups. Nitrogen concentrations increased over time in all treatments; W showed the highest levels, H intermediate levels, and T the lowest. Water exchange reduced concentrations in all groups, while the relative pattern (W > H > T) remained consistent.


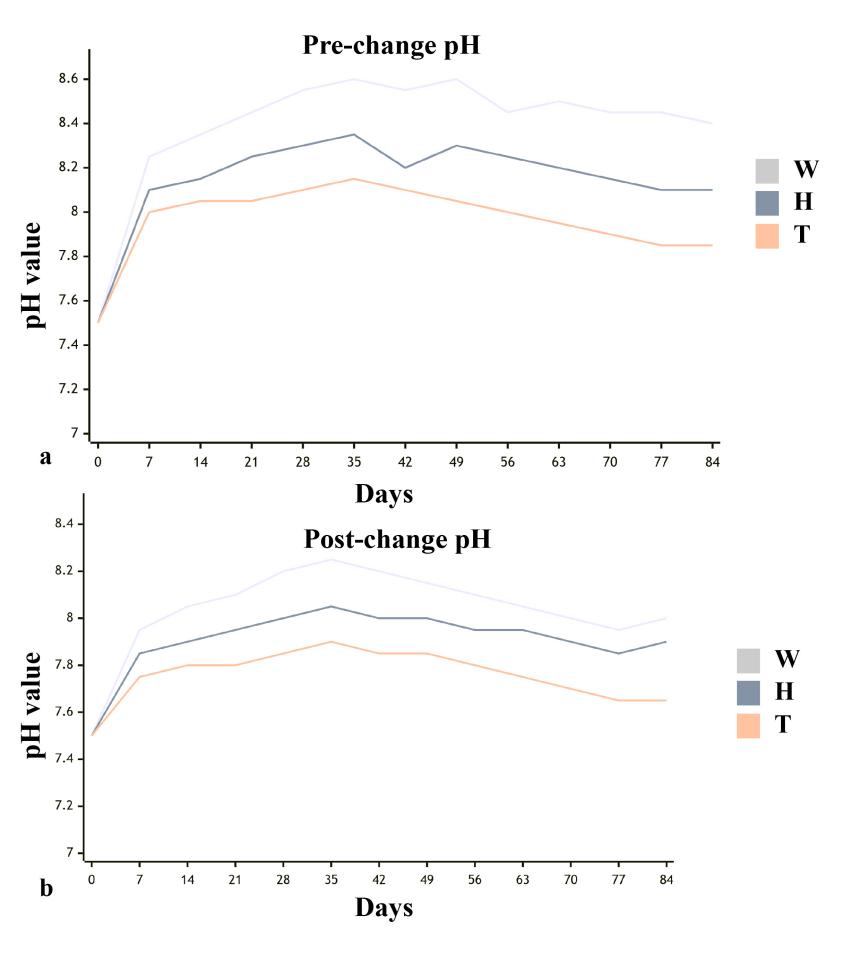


**Supplementary Figure S4.** Effect of 30% Water exchange on pH dynamics in three aquaculture treatment systems: (a) Pre-exchange pH: shows the progressive alkalinization during system operation, with all treatments starting at pH 7.5 and diverging to treatment-specific peaks (W: 8.6, H: 8.35, T: 8.15) due to metabolic accumulation. (b) Post-exchange pH : demonstrates the moderating effect of water exchange, showing reduced pH extremes (W: 8.25, H: 8.05, T: 7.9) and smoother trajectories while maintaining the consistent treatment hierarchy (W > H > T).


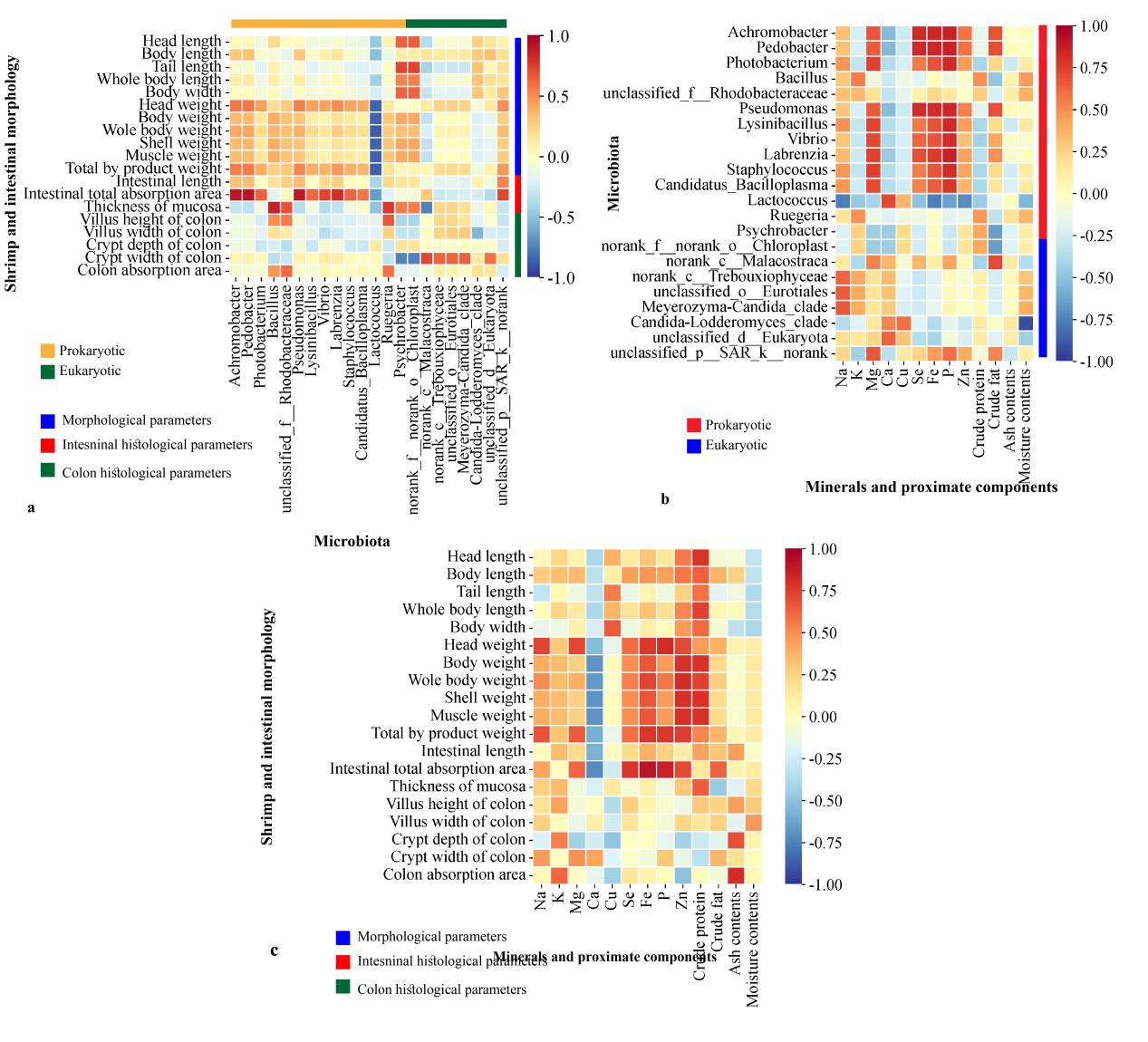


**Supplementary Figure S5.** **Correlation heatmaps investigating the relationships between shrimp biology and microbiome features.** The analyses explore correlations between: **(a)** Intestinal/hindgut morphology and microbiota composition; **(b)** Microbiota and meat composition (minerals and proximate components); **(c)** Histology and meat composition. Heatmaps employ a red-blue color gradient to represent the strength and direction (positive or negative) of Pearson correlation coefficients (*r*), categorized as follows: very strong (∣r∣>0.75∣*r*∣>0.75) to weak (∣r∣<0.25∣*r*∣<0.25).


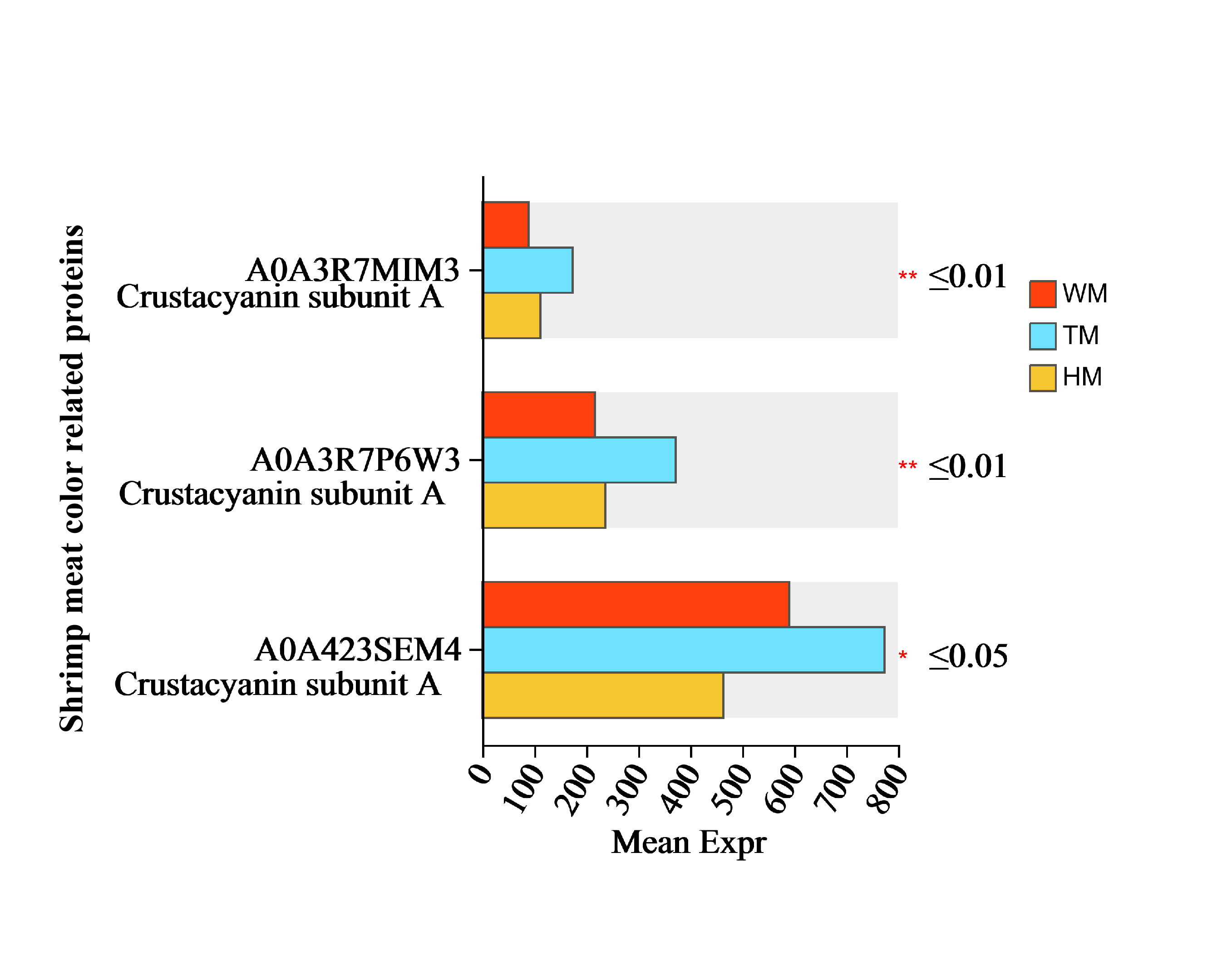


**Supplementary Figure S6.** Differential expression patterns of meat quality-associated proteins across experimental groups W, H, and T. Bar charts showing significant variations in differentially expressed proteins (DEPs) among the W, H, and T groups for shrimp meat color related proteins. The horizontal axis represents the mean relative protein expression across groups (WM, HM, TM; group name + meat sample), with different colored bars representing distinct groups. The vertical axis indicates protein designations. Red, blue, and orange markers correspond to groups W, H, and T, respectively. The p-value is indicated on the far right, where 0.01 < *p* ≤ 0.05, * 0.001< *p* ≤ 0.01, *** *p* ≤ 0.001.
