# Supplementary material for: Lactobacillus salivarius Probiotic Supplementation Modulates Gut Function, Improves Growth, and Meat Quality in Tropical Whiteleg Shrimp
Source: Aquac Nutr. 2026 May 27;2026:6285997. doi: 10.1155/anu/6285997 (PMC13213333; doi:10.1155/anu/6285997)
Supplement: Supplementary file 2 — Supporting Information 2 Note S1: Water Quality Testing Method. Note S2. Probiotic sources, preparation and application of commercial EM probiotic. and Lactobacillus salivarius strain GZPH2 for shrimp feed supplementation. Note S3. Growth performance and yield parameters related formulas. Note S4. pH Measurement of Shrimp Meat. Note S5. Meat Color Measurement (Raw and Boiled Shrimp). [file ANU-2026-6285997-s002.docx]

**Supplementary Note 1. Water Quality Testing Method**

Water quality parameters were monitored throughout the 90‑day experimental period to assess the culture environment. Sampling was performed at 7‑day intervals beginning on day 0. On each sampling day, 100 mL water samples were collected from each pond: one prior to water exchange and one immediately after exchanging 30% of the pond water. This sampling routine continued until harvest. pH, water temperature, and dissolved oxygen (DO) were measured directly on‑site using calibrated portable meters (pH meter, thermometer, and DO meter). For chemical analysis, additional 100 mL water samples were collected from each replicate pond at the same time points. All samples were stored on ice and transported to the field laboratory within 2 hours. Concentrations of ammonia‑nitrogen (NH₃‑N), nitrite‑nitrogen (NO₂⁻‑N), and nitrate‑nitrogen (NO₃⁻‑N) were determined using Zerui reagent test kits following the manufacturer's protocols.

**Supplementary Note 2. Probiotic sources, preparation and application of commercial EM probiotic and *Lactobacillus salivarius* strain GZPH2 for shrimp feed supplementation**

**Commercial Effective Microorganisms (EM) Probiotic**

**Probiotic source:** A commercially available EM probiotic concentrate powder was obtained from Shandong Jida Biotechnology Co., Ltd. (production license: Lu Si Tian (2021) H14543). The product consists of a multi-species microbial consortium, including *Lactobacillus* spp., *Bacillus* spp., nitrifying bacteria, *Actinobacteria*, and photosynthetic bacteria, with a declared viable count of at least 1.0 × 10¹¹ CFU/g.

**Preparation of fermentation medium:** To activate the dormant microorganisms, a fermentation mixture was prepared by combining 200 g of EM probiotic powder with 500 g of brown sugar as a carbon source. This mixture was dissolved thoroughly in 20 L of dechlorinated freshwater.

**Anaerobic activation process:** The prepared solution was transferred into a sealed fermentation container to establish anaerobic conditions. Fermentation was conducted at a controlled temperature of 28–30°C for a duration of 5 days without agitation.

**Verification of activated probiotic solution:** Upon completion of fermentation, the activated probiotic solution (APS) exhibited a mildly acidic pH of approximately 4.0, indicating successful microbial activation and lactic acid production.

**Storage and shelf life:** The APS was stored at ambient temperature and used within three weeks of preparation to ensure microbial viability and functional efficacy.

**Feed supplementation:** Prior to feeding, the APS was mixed uniformly with commercial shrimp feed at a dosage rate of 1 kg APS per 50 kg of feed. The treated feed was prepared fresh before use to minimize loss of probiotic activity.

**Experimental Probiotic *Lactobacillus salivarius* Strain GZPH2**

**Strain origin and identification:** *Lactobacillus salivarius* strain GZPH2, protected under Chinese Patent ZL201410752144.2, was used as the experimental probiotic. The strain was originally isolated from a commercially available pickle sample collected in Guangzhou, China. Molecular identification was performed using 16S rDNA sequencing (sequence data available at: https://wenju.baidu.com/view/2d5d8eff6e175f0e7cd184254b35eefdc9d315ef), and the strain was deposited in the China Type Culture Collection (CCTCC M 2014598).

**Safety assessment:** To confirm biosafety for aquatic application, the strain was evaluated for hemolytic activity on goat blood agar plates and was verified to be non-hemolytic (data not shown).

**Revival of frozen culture:** A cryopreserved vial of *Lactobacillus salivarius* strain GZPH2 was thawed and reactivated following a modified protocol based on Chinese Patent ZL202310736625.3. The strain was initially inoculated into de Man, Rogosa, and Sharpe (MRS) broth and incubated under static conditions.

**Purification of bacterial culture:** To ensure culture purity, the revived bacteria were streaked and sub-cultured three consecutive times on MRS agar plates until uniform colony morphology was observed.

**Preparation of culture medium:** The MRS medium was formulated per liter as follows: glucose (40.0 g), peptone (20.0 g), beef extract powder (20.0 g), yeast extract (20.0 g), sodium acetate (20.0 g), K₂HPO₄ (8.0 g), diammonium hydrogen citrate (8.0 g), NaCl (15.0 g), MgSO₄·7H₂O (0.4 g), MnSO₄·7H₂O (1.0 g), and Tween-80 (1 mL). The pH was adjusted to 6.5 ± 0.1 prior to sterilization. For solid media, bacteriological agar was added at 15.0 g/L. Media were autoclaved at 121°C and 0.1 MPa for 20 minutes.

**Seed stock preparation:** A seed culture was generated by inoculating 1 mL of the purified bacterial suspension into 50 mL of sterile MRS broth. The culture was incubated statically at 37°C for 24 hours.

**Enumeration of viable bacteria:** Bacterial density was quantified using serial dilution followed by spread plating on MRS agar. Plates containing 30–300 colonies were counted, and results were expressed as CFU/mL. The seed culture reached an approximate concentration of 8 × 10⁸ CFU/mL.

**Storage of seed culture:** The seed stock was stored at 4°C and used as the inoculum for subsequent probiotic production.

**Preparation of fresh probiotic culture:** For feed supplementation, 20 mL of seed stock was inoculated into 1000 mL of MRS broth and incubated statically at 37°C for 24 hours to obtain an actively growing bacterial culture.

**Feed coating procedure:** The freshly cultured probiotic suspension was mixed thoroughly with commercial shrimp feed at a volume-to-weight ratio of 1:3 (v/w). The coated feed was spread evenly in a thin layer and air-dried at 37°C for 1–3 hours to reduce surface moisture without compromising bacterial viability.

**Storage and usage:** Probiotic-supplemented feed was prepared twice per week, sealed in plastic bags, and stored at 4°C until use to maintain stability and efficacy.

**Supplementary Note 3. Growth performance and yield parameters related formulas:**

1. **Total weight gain (TWG)**
2. **Meat yield**

$$\text{Meat}\text{ }\text{yield}\text{ (}\text{g}\text{)=}\frac{\text{Meat}\text{ }\text{weig}\text{h}\text{t}\text{ (}\text{g}\text{)}}{\text{Body}\text{ }\text{weig}\text{h}\text{t}\text{ (}\text{g}\text{)}}\text{ ×100}$$

3. **By-product yield**

$$\text{By}\text{−}\text{product}\text{ }\text{yield}\text{ (}\text{g}\text{)= }\frac{\text{Total}\text{ }\text{by}\text{−}\text{oroduct}\text{ }\text{weig}\text{h}\text{t}\text{ (}\text{g}\text{)}}{\text{Body}\text{ }\text{weig}\text{h}\text{t}\text{ (}\text{g}\text{)}}\text{ ×100}$$

4. **Meat: waste ratio**

$$\text{Meat}\text{: }\text{waste}\text{ }\text{ratio}\text{ =}\frac{\text{Meat}\text{ }\text{weig}\text{h}\text{t}}{\text{Total}\text{ }\text{by}\text{−}\text{product}\text{ }\text{weig}\text{h}\text{t}}\text{ }$$

5. **Average daily gain (ADG)**

$$\text{ADG}\text{ (}\text{g day⁻¹}\text{)}\text{=}\frac{\text{Final}\text{ }\text{body}\text{ }\text{weig}\text{h}\text{t}\text{ (}\text{g}\text{) − }\text{Initial}\text{ }\text{body}\text{ }\text{weig}\text{h}\text{t}\text{ (}\text{g}\text{)}}{\text{Number}\text{ }\text{of}\text{ }\text{days}}$$

6. **Specific growth rate (SGR)**

$$\text{SGR}\text{ (}\text{\% day⁻¹}\text{)}\text{=}\frac{\text{In}\text{ (}\text{Final}\text{ }\text{body}\text{ }\text{weig}\text{h}\text{t}\text{)−}\text{In}\text{(}\text{Initial}\text{ }\text{body}\text{ }\text{weig}\text{h}\text{t}\text{)}}{\text{Numbers}\text{ }\text{of}\text{ }\text{days}}\text{×100}$$

Where In= natural logarithm

1. **Total biomass gain (TBG)**

$$\text{TBG}\text{ (}\text{Kg}\text{)= }\frac{\text{TWG}\text{ (}\text{g}\text{)×}\text{Number}\text{ }\text{of}\text{ }\text{harvested shrimp}}{1000}$$

Here, TWG = Final body weight - Initial body weight, To convert to kg: divide by 1000

1. **Feed conversion ratio (FCR)**

$$\text{FCR}\text{=}\frac{\text{Total}\text{ }\text{feed}\text{ }\text{input}}{\text{Total}\text{ }\text{biomass}\text{ }\text{gain}}$$

2. **Survival rate**

$$\text{Survival}\text{ }\text{rate}\text{ (\%)=}\frac{\text{Final}\text{ }\text{number}\text{ }\text{of}\text{ }\text{s}\text{h}\text{rimp}}{\text{Initial}\text{ }\text{number}\text{ }\text{of}\text{ }\text{s}\text{h}\text{rimp}}\text{×100}$$

### ****Supplementary Note 4. pH Measurement of Shrimp Meat****

Shrimp meat pH was measured using a digital pH meter (**HI 2211 pH Meter, Hanna Instruments, USA**). The instrument was calibrated prior to use with standard buffer solutions (pH 4.0, 7.0, and 10.0) according to the manufacturer’s instructions.

For each treatment group (W, H, and T), **three live shrimp were randomly selected from each replicate**. The shrimp were placed on an ice tray for 2–3 minutes to minimize stress prior to processing. The shells were then removed, and the muscle tissue was carefully collected. pH measurements were taken from **three different locations of the muscle**, and the mean value was recorded for each sample.

To assess post-mortem changes, shrimp meat samples were subsequently kept at **ambient temperature**, and pH was measured at **6-hour intervals** over a 24-hour period. The post-mortem pH decline followed the pattern **W > H > T**, indicating higher glycolytic activity and lactic acid accumulation in the control group compared to probiotic-treated groups.

pH changed during post-mortem storage. Supplementary Table S8 shows that initial pH at 0 h was similar across groups (7.20–7.24). Over 24 h, pH declined in all groups, but the mixed probiotic T group consistently maintained the highest pH values at all time points (6.10 at 24 h), followed by H (6.07) and W (5.91), indicating slower post-mortem acidification in probiotic-treated shrimp.

### ****Supplementary Note 5. Meat Color Measurement (Raw and Boiled Shrimp)****

Shrimp meat color was measured using a portable colorimeter (Minolta CR-400/CR-410, Konica Minolta, Japan) based on the CIELAB color system, which expresses color as lightness (*L**), redness (*a**), and yellowness (*b**). The instrument was calibrated using a standard white calibration plate prior to measurement according to the manufacturer’s instructions.

For each treatment group (W, H, and T), three healthy shrimp were randomly selected from each replicate (WS1–WS3, HS1–HS3, TS1–TS3). For **raw color measurement**, peeled shrimp muscle was placed on a flat surface, and readings were taken from the dorsal region under consistent lighting conditions.

For **boiled samples**, shrimp were cooked in boiling water (100 °C) for 2–3 minutes until fully cooked, then immediately cooled to room temperature. The shells were removed, and the muscle was gently blotted to remove surface moisture before measurement. Color readings were then taken from the same dorsal region under identical conditions. For both raw and boiled samples, measurements were recorded at three different positions per sample, and the mean value was used for analysis.

Color parameters were defined as follows: *L** represents lightness (0 = black, 100 = white), *a** indicates redness (positive values) to greenness (negative values), and *b** indicates yellowness (positive values) to blueness (negative values). This method provides an objective evaluation of shrimp meat appearance and is widely used to assess pigment stability, cooking-induced color changes, and overall product quality.
