# Supplementary material for: Lactobacillus salivarius Probiotic Supplementation Modulates Gut Function, Improves Growth, and Meat Quality in Tropical Whiteleg Shrimp
Source: Aquac Nutr. 2026 May 27;2026:6285997. doi: 10.1155/anu/6285997 (PMC13213333; doi:10.1155/anu/6285997)
Supplement: Supplementary file 3 — Supporting Information 3 Table S1: The nutritional composition of feed (In supporting tables file: page 1). Table S2. KEGG‐based profiling of absorption‐related pathways in intestinal microbiota across treatments W, H, and T (In supporting tables file: start from page 1 continue to page 4). Table S3. KEGG‐based annotation of mineral (ion) metabolism and transport in intestinal microbiota across treatments W, H, and T (In supporting tables file: start from page 4 continue to page 5). Table S4. KEGG‐based annotation of metabolism‐related enzymes in intestinal microbiota across treatments W, H, and T (In supporting tables file: start from page 6 continue to page 9). Table S5. KEGG‐based annotation of immunity‐related enzymes in intestinal microbiota across treatments W, H, and T (In supporting tables file: start from page 9 continue to page 12). Table S6. KEGG‐based annotation of toxin‐related (virulence‐associated) genes in intestinal microbiota across treatments W, H, and T (In supporting tables file: start from page 12 continue to page 13). Table S7. KEGG‐based annotation of adhesion, colonization, and secretion system‐related genes in intestinal microbiota across treatments W, H, and T (In supporting tables file: start from page 13 continue to page 14). Table S8. Post‐mortem pH changes of shrimp meat (0–24 h) across treatments W, H, and T (In supporting tables file: page 14). Table S9. Full protein functional annotation table (In supporting tables file: page 15). Table S10. Statistical table of the number of differential proteins in shrimp meat across treatments W, H, and T (In supporting tables file: page 15). Table S11. Protein abundance of major structural, calcium‑binding, and oxygen‑transport proteins in shrimp meat across group W, H, and T (In supporting tables file: start from page 15 continue to page 17). Table S12. Differential expression of carbohydrate metabolism enzymes and pathways in shrimp muscle across group W, H, and T (In supporting tables file: s [file ANU-2026-6285997-s001.docx]

**Supplementary Table S1.** The nutritional composition of feed

| Nutrient | Composition |
| --- | --- |
| Crude Protein | ≥ 42% |
| Carbohydrates | ≥ 10.75% |
| Calcium (Ca) | ≤ 4% |
| Sodium Chloride (NaCl) | ≤ 3% |
| Lysine | > 2.25% |
| Crude Fat | ≥ 4% |
| Crude Fibre | ≤ 5% |
| Total Phosphorus | ≥ 1% |
| Crude Ash Content | ≤ 16% |
| Moisture | ≤ 12% |

**Supplementary Table S2.** KEGG-based profiling of absorption-related pathways in intestinal microbiota across treatments W, H, and T

| KO ID | KO Description | Pathway | Transport Type | WI | HI | TI | Dominant Group |
| --- | --- | --- | --- | --- | --- | --- | --- |
| K01995 | branched-chain amino acid transport system ATP-binding protein | Amino acid transport | Branched-chain AA uptake | 40728.34 ± 0 | 187550.16 ± 0 | 21181.89 ± 0 | HI |
| K01996 | branched-chain amino acid transport system ATP-binding protein | Amino acid transport | Branched-chain AA uptake | 40867.84 ± 0 | 188039.16 ± 0 | 21219.39 ± 0 | HI |
| K01997 | branched-chain amino acid transport system permease protein | Amino acid transport | Branched-chain AA uptake | 40618.84 ± 0 | 186483.66 ± 0 | 21198.39 ± 0 | HI |
| K01998 | branched-chain amino acid transport system permease protein | Amino acid transport | Branched-chain AA uptake | 40203.84 ± 0 | 185593.66 ± 0 | 21206.39 ± 0 | HI |

**Note:** Data are mean ± SD (n = 3 replicate ponds per treatment: WS1–WS3, HS1–HS3, TS1–TS3). Different superscript letters in the same column denote statistically significant differences (*p* < 0.05), while identical letters indicate no significant differences (*p* > 0.05). Abbreviations: WI, HI, and TI denote the intestinal samples from groups W, H, and T, respectively.

**Supplementary Table S2** (**continued**). KEGG-based profiling of absorption-related pathways in intestinal microbiota across treatments W, H, and T

| **KO ID** | **KO Description** | **Pathway** | **Transport Type** | **WI** | **HI** | **TI** | **Dominant Group** |
| --- | --- | --- | --- | --- | --- | --- | --- |
| K01999 | branched-chain amino acid transport system substrate-binding protein | Amino acid transport | Branched-chain AA uptake | 31602.42 ± 7604.65 | 157333.41 ± 37522.66 | 19826.84 ± 1966.25 | HI |
| K02030 | polar amino acid transport system substrate-binding protein | Amino acid transport | Polar AA uptake | 175010.76 ± 13991.42 | 177673.33 ± 27665.56 | 66179.65 ± 28132.66 | HI |
| K02028 | polar amino acid transport system ATP-binding protein [EC:7.4.2.1] | Amino acid transport | Polar AA uptake | 102199.1 ± 81.45 | 180399.41 ± 123.5 | 42693.34 ± 167.5 | HI |
| K02029 | polar amino acid transport system permease protein | Amino acid transport | Polar AA uptake | 105796.71 ± 1245.9 | 190442.1 ± 1678.3 | 43809.8 ± 1342.1 | HI |
| K02424 | L-cystine transport system substrate-binding protein | Amino acid transport | Cystine uptake | 4327.5 ± 12.9 | 18120.3 ± 1.99 | 22308.21 ± 29.98 | TI |
| K10036 | glutamine transport system substrate-binding protein | Amino acid transport | Glutamine uptake | 3297 ± 13.2 | 16866 ± 1.98 | 63.71 ± 1.02 | HI |
| K10037 | glutamine transport system permease protein | Amino acid transport | Glutamine uptake | 3297 ± 81.23 | 16866 ± 331.11 | 63.71 ± 1.78 | HI |
| K10038 | glutamine transport system ATP-binding protein [EC:7.4.2.1] | Amino acid transport | Glutamine uptake | 3297 ± 821.9 | 16900 ± 2145.8 | 70.71 ± 12.8 | HI |
| K03320 | ammonium transporter, Amt family | Ammonium transport | Ammonium uptake | 8086.16 ± 5691.74 | 34460.19 ± 31092.18 | 22054.87 ± 30894.89 | HI |

**Note:** Data are mean ± SD (n = 3 replicate ponds per treatment: WS1–WS3, HS1–HS3, TS1–TS3). Different superscript letters in the same column denote statistically significant differences (*p* < 0.05), while identical letters indicate no significant differences (*p* > 0.05). Abbreviations: WI, HI, and TI denote the intestinal samples from groups W, H, and T, respectively.

**Supplementary Table S2** (**continued**). KEGG-based profiling of absorption-related pathways in intestinal microbiota across treatments W, H, and T

| **KO ID** | **KO Description** | **Pathway** | **Transport Type** | **WI** | **HI** | **TI** | **Dominant Group** |
| --- | --- | --- | --- | --- | --- | --- | --- |
| K02010 | Fe³⁺ transport system ATP-binding protein [EC:7.2.2.7] | Iron transport | Iron uptake | 25976.56 ± 18537.09 | 126998.55 ± 120604.28 | 42511.93 ± 48556.39 | HI |
| K02011 | Fe³⁺ transport system permease protein | Iron transport | Iron uptake | 25990.06 ± 19032.39 | 128925.35 ± 121194.28 | 42512.33 ± 48556.39 | HI |
| K02012 | Fe³⁺ transport system substrate-binding protein | Iron transport | Iron uptake | 27217.23 ± 18982.89 | 130125.24 ± 123958.78 | 62932.83 ± 66372.64 | HI |
| K02013 | iron complex transport system ATP-binding protein [EC:7.2.2.-] | Iron transport | Iron uptake | 101628.51 ± 93397.96 | 176154.86 ± 191011.2 | 125663.01 ± 132493.59 | HI |
| K02014 | iron complex outermembrane recepter protein | Iron transport | Iron uptake | 47855.74 ± 37703.99 | 245083.24 ± 220937.34 | 5923.8 ± 39731.87 | HI |
| K02015 | iron complex transport system permease protein | Iron transport | Iron uptake | 107167.12 ± 97593.06 | 201832.82 ± 229011.74 | 209422.02 ± 228817.26 | TI |
| K02016 | iron complex transport system substrate-binding protein | Iron transport | Iron uptake | 66954.33 ± 57664.74 | 163006.05 ± 186992.7 | 208161.81 ± 216190.09 | TI |
| K07230 | periplasmic iron binding protein | Iron transport | Iron uptake | 3283.33 ± 2395.33 | 16865 ± 16044 | 20450.1 ± 17838.25 | TI |
| K09815 | Zn²⁺ transport system substrate-binding protein | Metal transport | Zinc uptake | 34841.44 ± 35361.91 | 9204.96 ± 8814.78 | 21596.93 ± 30446.39 | WI |
| K09817 | Zn²⁺ transport system ATP-binding protein [EC:7.2.2.-] | Metal transport | Zinc uptake | 34786.94 ± 35361.91 | 8360.96 ± 8751.28 | 21601.76 ± 30444.14 | WI |
| K02074 | Zn²⁺ / Mn²⁺ transport system ATP-binding protein | Metal transport | Metal uptake | 3575.33 ± 2706.61 | 17494 ± 16631 | 110.71 ± 83.14 | HI |

**Note:** Data are mean ± SD (n = 3 replicate ponds per treatment: WS1–WS3, HS1–HS3, TS1–TS3). Different superscript letters in the same column denote statistically significant differences (*p* < 0.05), while identical letters indicate no significant differences (*p* > 0.05). Abbreviations: WI, HI, and TI denote the intestinal samples from groups W, H, and T, respectively.

**Supplementary Table S2** (**continued**). KEGG-based profiling of absorption-related pathways in intestinal microbiota across treatments W, H, and T

| **KO ID** | **KO Description** | **Pathway** | **Transport Type** | **WI** | **HI** | **TI** | **Dominant Group** |
| --- | --- | --- | --- | --- | --- | --- | --- |
| K02075 | Zn²⁺ / Mn²⁺ transport system permease protein | Metal transport | Metal uptake | 3659.95 ± 3136.94 | 18175.63 ± 16866.74 | 158.71 ± 89.14 | HI |
| K02077 | zinc/manganese transport system substrate-binding protein | Metal transport | Metal uptake | 3710.28 ± 3195.94 | 18336.63 ± 16907.74 | 162.71 ± 95.14 | HI |
| K01008 | selenide, water dikinase [EC:2.7.9.3] | Selenocysteine biosynthesis | Selenocysteine synthesis | 3429.16 ± 861.3 | 20539.98 ± 3338.05 | 151.66 ± 82.07 | HI |
| K10764 | O-succinylhomoserine sulfhydrylase [EC:2.5.1.-] | Selenocysteine biosynthesis | Selenocysteine biosynthesis | 1685.08 ± 676.47 | 7159.07 ± 2262.66 | 6868.42 ± 8134.64 | HI |
| K11996 | adenylyltransferase and sulfurtransferase [EC:2.7.7.80 2.8.1.11] | Sulfur/selenium transfer | Selenium transfer | 2736.39 ± 847.31 | 20537.23 ± 3833.85 | 64355.77 ± 2550.69 | TI |

**Note:** Data are mean ± SD (n = 3 replicate ponds per treatment: WS1–WS3, HS1–HS3, TS1–TS3). Different superscript letters in the same column denote statistically significant differences (*p* < 0.05), while identical letters indicate no significant differences (*p* > 0.05). Abbreviations: WI, HI, and TI denote the intestinal samples from groups W, H, and T, respectively.

**Supplementary Table S3.** KEGG-based annotation of mineral (ion) metabolism and transport in intestinal microbiota across treatments W, H, and T

| **Enzyme** | **WI** | **HI** | **TI** | **Dominant Group** | **Role** |
| --- | --- | --- | --- | --- | --- |
| Ferroxidase (EC 1.16.3.1) | 6,881.75 ± 1,489.47 | 33,222.97 ± 7,279.50 | 33,021.49 ± 14,379.53 | HI/TI | Iron (Fe) oxidation/transport |
| Iron-chelate-transporting ATPase (EC 3.6.3.34) | 100,289.48 ± 5,184.76 | 173,913.61 ± 17,091.32 | 129,078.30 ± 4,843.13 | HI | Iron (Fe) import |
| Fe(3+)-transporting ATPase (EC 3.6.3.30) | 20,994.87 ± 4,428.62 | 116,506.27 ± 9,859.57 | 45,554.41 ± 3,184.08 | HI | Iron (Fe) transport |
| Cu(2+)-exporting ATPase (EC 3.6.3.4) | 35,356.10 ± 2,004.71 | 13,568.67 ± 4,804.60 | 12,787.95 ± 9,682.98 | WI | Copper (Cu) export |

**Note:** Data are mean ± SD (n = 3 replicate ponds per treatment: WS1–WS3, HS1–HS3, TS1–TS3). Different superscript letters in the same column denote statistically significant differences (*p* < 0.05), while identical letters indicate no significant differences (*p* > 0.05). Abbreviations: WI, HI, and TI denote the intestinal samples from groups W, H, and T, respectively.

**Supplementary Table S3** (**continued**). KEGG-based annotation of mineral (ion) metabolism and transport in intestinal microbiota across treatments W, H, and T

| **Enzyme** | **WI** | **HI** | **TI** | **Dominant Group** | **Role** |
| --- | --- | --- | --- | --- | --- |
| Zinc/Cadmium-exporting ATPase 3.6.3.5/3 | 38,102.72 ± 2,453.98 | 31,856.23 ± 5,373.69 | 45,107.31 ± 4,322.13 | TI | Zinc (Zn) / Cadmium (Cd) export |
| Calcium-transporting ATPase (EC 3.6.3.8) | 33,022.73 ± 2,149.61 | 6,410.20 ± 4,514.87 | 38,369.55 ± 1,656.12 | TI | Calcium (Ca) transport |
| H(+)-transporting two-sector ATPase (EC 3.6.3.14) | 86,669.37 ± 3,673.56 | 101,286.63 ± 19,782.52 | 71,669.53 ± 10,403.72 | HI | Proton (H+) gradient / pH |
| Phosphate-transporting ATPase (EC 3.6.3.27) | 72,176.90 ± 6,422.94 | 30,848.25 ± 6,702.12 | 26,197.50 ± 6,192.20 | WI | Phosphate (Pi) import |
| Monosaccharide-transporting ATPase (EC 3.6.3.17) | 80,755.77 ± 6,075.64 | 48,446.81 ± 4,470.79 | 38,524.13 ± 3,822.58 | WI | Nutrient import (coupled with ions) |
| Magnesium-importing ATPase (EC3.6.3.2 ) | 35,607.22 ± 2,312.39 | 18,835.94 ± 6,239.78 | 19,503.79 ± 1,110.63 | WI | Magnesium (Mg) import |
| Nickel-transporting ATPase (EC 3.6.3.24) | 19.34 ± 19.12 | 658.44 ± 617.45 | 0.02 ± 0.02 | HI | Nickel (Ni) import |
| Arsenate reductase (glutaredoxin) (EC 1.20.4.1) | 78,859.08 ± 5,894.98 | 59,792.71 ± 1,351.03 | 45,565.30 ± 2,643.64 | WI | Arsenic (As) detoxification |
| Molybdopterin adenylyltransferase (EC 2.7.7.75) | 2,991.87 ± 506.97 | 17,325.22 ± 738.01 | 6,428.93 ± 5,577.29 | HI | Molybdenum (Mo) cofactor biosynthesis |
| Ferric-chelate reductase (NADPH) (EC 1.16.1.9) | 0.01 ± 0.01 | 0.56 ± 0.96 | 2.85 ± 2.02 | TI | Iron (Fe) reduction/uptake |
| Molybdate-transporting ATPase (EC 3.6.3.29) | 3,863.98 ± 921.90 | 21,186.90 ± 2,469.99 | 339.54 ± 116.16 | HI | Molybdenum (Mo) import |
| Tungstate-importing ATPase (EC 3.6.3.55) | 597.21 ± 302.85 | 3,174.09 ± 1,774.37 | 52.79 ± 60.55 | HI | Tungstate (W) import |
| Heme oxygenase (staphylobilin-producing) (EC 1.14.99.48) | 165.67 ± 223.68 | 4,183.00 ± 5,961.86 | 38,234.05 ± 3,656.46 | TI | Heme (Iron) degradation |
| Ferrochelatase (EC 4.99.1.1) | 5,357.99 ± 1,363.63 | 30,273.33 ± 1,545.29 | 42,299.89 ± 4,235.40 | TI | Iron insertion into porphyrin (heme synthesis) |
| Magnesium chelatase (EC 6.6.1.1) | 1,668.23 ± 716.57 | 7,457.97 ± 2,313.25 | 112.25 ± 49.33 | HI | Magnesium insertion into chlorophyll (bacterial/plant) |

**Note:** Data are mean ± SD (n = 3 replicate ponds per treatment: WS1–WS3, HS1–HS3, TS1–TS3). Different superscript letters in the same column denote statistically significant differences (*p* < 0.05), while identical letters indicate no significant differences (*p* > 0.05). Abbreviations: WI, HI, and TI denote the intestinal samples from groups W, H, and T, respectively.

**Supplementary Table S4.** KEGG-based annotation of metabolism-related pathways and enzymes in intestinal microbiota across treatments W, H, and T

| **KO ID** | **KEGG Pathway** | **Enzyme** | **WI** | **HI** | **TI** | **Functional Role** | **Dominant Group** |
| --- | --- | --- | --- | --- | --- | --- | --- |
| K00134 | Glycolysis | Glyceraldehyde-3-phosphate dehydrogenase (EC 1.2.1.12) | 44,288.36 ± 2,774.12 | 51,703.32 ± 3,313.42 | 52,301.52 ± 12,352.56 | NADH production | TI |
| K00873 | Glycolysis | Pyruvate kinase (EC 2.7.1.40) | 41,114.72 ± 3,287.24 | 36,495.54 ± 6,508.22 | 38,548.39 ± 4,094.19 | ATP production | WI |
| K00036 | Pentose phosphate pathway | Glucose-6-phosphate dehydrogenase (EC 1.1.1.49) | 37,704.63 ± 3,194.82 | 15,063.46 ± 2,766.85 | 38,396.46 ± 3,803.27 | NADPH production | TI |
| K00033 | Pentose phosphate pathway | 6-phosphogluconate dehydrogenase (EC 1.1.1.44) | 73,542.95 ± 6,337.65 | 14,561.48 ± 3,069.76 | 57,492.31 ± 5,589.41 | Oxidative phase of the Pentose Phosphate Pathway (PPP) | WI |
| K01689 | Glycolysis | Enolase (EC 4.2.1.11) | 42,732.00 ± 2,730.46 | 44,744.83 ± 4,108.76 | 47,721.59 ± 6,242.30 | Phosphoenolpyruvate Formation (PEP) formation | TI |
| K01223 | Glycolysis | 6-phospho-beta-glucosidase | 7013.5 ± 6595.9 | 94.2 ± 0.0 | 241253.3 ± 10507.0 | hydrolysis of phosphorylated β-glucosides ( cellobiose-6-phosphate) into glucose-6-phosphate and an aglycone | WI |
| K00099 | Terpenoid biosynthesis | DXP reductoisomerase (EC 1.1.1.267) | 5,883.42 ± 1,293.84 | 29,876.07 ± 2,780.91 | 45,272.38 ± 4,377.14 | Isoprenoid synthesis | TI |

**Note:** Data are mean ± SD (n = 3 replicate ponds per treatment: WS1–WS3, HS1–HS3, TS1–TS3). Different superscript letters in the same column denote statistically significant differences (*p* < 0.05), while identical letters indicate no significant differences (*p* > 0.05). Abbreviations: WI, HI, and TI denote the intestinal samples from groups W, H, and T, respectively.

**Supplementary Table S4**(**continued**)**.** KEGG-based annotation of metabolism-related pathways and enzymes in intestinal microbiota across treatments W, H, and T

| **KO ID** | **KEGG Pathway** | **Enzyme** | **WI** | **HI** | **TI** | **Functional Role** | **Dominant Group** |
| --- | --- | --- | --- | --- | --- | --- | --- |
| K00208 | Fatty acid biosynthesis | Enoyl-ACP reductase (NADH) (EC 1.3.1.9) | 52,135.34 ± 3,311.59 | 91,758.98 ± 8,104.29 | 26,418.55 ± 6,128.49 | Fatty acid (FA) elongation | HI |
| K00059 | Fatty acid biosynthesis | 3-oxoacyl-ACP reductase (EC 1.1.1.100) | 147,333.72 ± 7,704.10 | 221,159.55 ± 30,720.47 | 174,881.78 ± 9,224.49 | FA synthesis | HI |
| K00626 | Fatty acid metabolism | Acetyl-CoA acetyltransferase (EC 2.3.1.9) | 64,819.43 ± 5,310.06 | 151,208.51 ± 22,635.71 | 92,124.01 ± 34,793.73 | Lipid metabolism | HI |
| K10224 | Fatty acid biosynthesis | Stearoyl-CoA desaturase (EC 1.14.19.1) | 4,084.53 ± 468.49 | 24,349.93 ± 2,152.28 | 7,158.33 ± 8,064.37 | FA desaturation | HI |
| K01652 | BCAA biosynthesis | Acetolactate synthase (EC 2.2.1.6) | 70,120.61 ± 7,012.06 | 183,794.27 ± 12,379.47 | 117,084.11 ± 14,872.37 | Branched-chain amino acid (AA) synthesis | HI |
| K00058 | Serine metabolism | Phosphoglycerate dehydrogenase (EC 1.1.1.95) | 19,761.98 ± 4,372.57 | 103,977.27 ± 17,208.64 | 26,477.12 ± 6,189.94 | Serine biosynthesis | HI |
| K00019 | Valine metabolism | 3-hydroxyisobutyrate dehydrogenase (EC 1.1.1.31) | 48,133.43 ± 3,011.50 | 65,406.39 ± 9,161.22 | 45,690.08 ± 4,490.91 | Valine catabolism | HI |
| K01733 | Isoleucine biosynthesis | Threonine ammonia-lyase (EC 4.3.1.19) | 53,168.39 ± 4,213.98 | 94,416.76 ± 15,594.54 | 46,682.78 ± 4,199.41 | Isoleucine Biosynthesis (Ile synthesis) | HI |
| K01695 | Tryptophan metabolism | Tryptophan synthase (EC 4.2.1.20) | 15,972.03 ± 3,219.88 | 89,514.14 ± 11,072.41 | 52,602.03 ± 12,425.70 | Tryptophan biosynthesis | HI |
| K00826 | Amino acid metabolism | BCAA transaminase (EC 2.6.1.42) | 46,386.71 ± 3,478.48 | 58,097.34 ± 6,545.39 | 45,407.56 ± 4,307.66 | Amino acid interconversion | HI |
| K01358 | Protein processing | Clp protease (EC 3.4.21.92) | 44,514.46 ± 3,274.67 | 51,255.92 ± 6,199.93 | 43,473.55 ± 2,908.65 | Protein degradation | HI |

**Note:** Data are mean ± SD (n = 3 replicate ponds per treatment: WS1–WS3, HS1–HS3, TS1–TS3). Different superscript letters in the same column denote statistically significant differences (*p* < 0.05), while identical letters indicate no significant differences (*p* > 0.05). Abbreviations: WI, HI, and TI denote the intestinal samples from groups W, H, and T, respectively.

**Supplementary Table S4** (**continued**)**.** KEGG-based annotation of metabolism-related pathways and enzymes in intestinal microbiota across treatments W, H, and T

| **KO ID** | **KEGG Pathway** | **Enzyme** | **WI** | **HI** | **TI** | **Functional Role** | **Dominant Group** |
| --- | --- | --- | --- | --- | --- | --- | --- |
| K01265 | Protein processing | Methionyl aminopeptidase (EC 3.4.11.18) | 44,658.07 ± 3,490.16 | 51,179.47 ± 7,716.96 | 47,647.77 ± 2,485.40 | Protein maturation | HI |
| K01868 | Translation | Asparaginyl-tRNA synthetase (EC 6.3.5.6) | 135,853.30 ± 10,414.33 | 166,997.34 ± 32,285.64 | 78,618.24 ± 18,829.44 | tRNA charging | HI |
| K00013 | Vitamin B6 metabolism | 4-hydroxythreonine phosphate dehydrogenase (EC 1.1.1.262) | 13,192.31 ± 2,570.73 | 70,540.95 ± 9,013.46 | 7,182.59 ± 8,057.55 | Pyridoxal 5′-phosphate (PLP) biosynthesis | HI |
| K03768 | Protein folding | Peptidylprolyl isomerase (EC 5.2.1.8) | 180,281.65 ± 10,344.13 | 231,225.16 ± 22,492.45 | 126,280.85 ± 48,894.63 | Protein folding | HI |
| K00230 | Porphyrin metabolism | Coproporphyrinogen dehydrogenase (EC 1.3.99.22) | 45,824.57 ± 2,518.40 | 64,269.09 ± 5,619.36 | 52,225.92 ± 12,403.80 | Heme biosynthesis | HI |
| K01845 | Porphyrin metabolism | Glutamate-1-semialdehyde aminomutase (EC 5.4.3.8) | 5,932.59 ± 1,374.84 | 35,803.80 ± 4,448.60 | 43,323.45 ± 4,353.42 | Tetrapyrrole biosynthesis | TI |
| K00425 | Energy Metabolism | Cytochrome bd ubiquinol oxidase subunit I [EC:7.1.1.7] | 46160.14 ± 2873.35 | 60124.24 ± 7479.77 | 64738.28 ± 2547.45 | Cytochrome bd oxidase - microaerophilic respiration, oxidative stress resistance | TI |
| K00426 | Energy Metabolism | Cytochrome bd ubiquinol oxidase subunit II [EC:7.1.1.7] | 46132.31 ± 2993.21 | 60668.58 ± 8047.95 | 83832.16 ± 701.22 | Cytochrome bd oxidase - microaerophilic respiration, oxidative stress resistance | TI |
| K02276 | Energy Metabolism | Cytochrome c oxidase subunit III [EC:7.1.1.9] | 5865.86 ± 1417.75 | 34068.8 ± 1959.12 | 19345.33 ± 1985.14 | Cytochrome c oxidase - aerobic respiration | HI |

**Note:** Data are mean ± SD (n = 3 replicate ponds per treatment: WS1–WS3, HS1–HS3, TS1–TS3). Different superscript letters in the same column denote statistically significant differences (*p* < 0.05), while identical letters indicate no significant differences (*p* > 0.05). Abbreviations: WI, HI, and TI denote the intestinal samples from groups W, H, and T, respectively.

**Supplementary Table S4**(**continued**)**.** KEGG-based annotation of metabolism-related pathways and enzymes in intestinal microbiota across treatments W, H, and T

| **KO ID** | **KEGG Pathway** | **Enzyme** | **WI** | **HI** | **TI** | **Role** | **Dominant Group** |
| --- | --- | --- | --- | --- | --- | --- | --- |
| K00239 | Energy Metabolism | Succinate dehydrogenase flavoprotein subunit [EC:1.3.5.1 1.3.5.4] | 6111.92 ± 1577.18 | 35206.06 ± 1506.2 | 26199.1 ± 6172.91 | Succinate dehydrogenase - TCA cycle, electron transport | HI |
| K01647 | Energy Metabolism | Citrate synthase [EC:2.3.3.1] | 49143.76 ± 3301.87 | 75433.6 ± 11161.1 | 45464.89 ± 4338.35 | Citrate synthase - TCA cycle, energy production | HI |
| K00016 | Lactic acid fermentation | L-lactate dehydrogenase [EC:1.1.1.27] | 109,479.54± 11,722.94 | 19,082.21±6,552.73 | 57,434.43±5,520.80 | Pyruvate to L-lactate (lactic acid production) | WI |

**Note:** Data are mean ± SD (n = 3 replicate ponds per treatment: WS1–WS3, HS1–HS3, TS1–TS3). Different superscript letters in the same column denote statistically significant differences (*p* < 0.05), while identical letters indicate no significant differences (*p* > 0.05). Abbreviations: WI, HI, and TI denote the intestinal samples from groups W, H, and T, respectively.

**Supplementary Table S5.** KEGG-based annotation of immunity-related pathways and enzymes in intestinal microbiota across treatments W, H, and T

| **KO ID** | **KO Description** | **Pathway Category** | **WI** | **HI** | **TI** | **Dominent group** | **Functional Role** |
| --- | --- | --- | --- | --- | --- | --- | --- |
| K01426 | Amidase [EC:3.5.1.4] | Host immune defense | 50988.13 ± 3804.11 | 80463.43 ± 21001.56 | 64593.89 ± 2668.13 | HI | Amidase - ammonia release, host defense |
| K01448 | N-acetylmuramoyl-L-alanine amidase [EC:3.5.1.28] | Host immune defense | 9751.19 ± 3158.03 | 57897.57 ± 14603.65 | 115161.4 ± 11148.78 | TI | N-acetylmuramoyl-L-alanine amidase - cell wall degradation |

**Note:** Data are mean ± SD (n = 3 replicate ponds per treatment: WS1–WS3, HS1–HS3, TS1–TS3). Different superscript letters in the same column denote statistically significant differences (*p* < 0.05), while identical letters indicate no significant differences (*p* > 0.05). Abbreviations: WI, HI, and TI denote the intestinal samples from groups W, H, and T, respectively.

**Supplementary Table S5 (continued).** KEGG-based annotation of immunity-related pathways and enzymes in intestinal microbiota across treatments W, H, and T

| **KO ID** | **KO Description** | **Pathway Category** | **WI** | **HI** | **TI** | **Dominent group** | **Functional Role** |
| --- | --- | --- | --- | --- | --- | --- | --- |
| K01449 | N-acetylmuramoyl-L-alanine amidase [EC:3.5.1.28] | Host immune defense | 724.67 ± 992.43 | 7275.47 ± 10824.5 | 95614.25 ± 9133.42 | TI | N-acetylmuramoyl-L-alanine amidase - cell wall degradation |
| K00128 | Aldehyde dehydrogenase (NAD+) [EC:1.2.1.3] | Detoxification | 14909.86 ± 5527.24 | 76526.21 ± 19900.77 | 71191.63 ± 10661.24 | HI | Aldehyde dehydrogenase - aldehyde detoxification |
| K00135 | Succinate-semialdehyde dehydrogenase [EC:1.2.1.16 1.2.1.79 1.2.1.20] | Detoxification | 14256.71 ± 3275.68 | 73564.06 ± 10435.85 | 39832.56 ± 22252.17 | HI | Succinate-semialdehyde dehydrogenase - GABA metabolism |
| K00265 | Glutamate synthase (EC 1.4.1.13/14) | Nitrogen assimilation | 11,570.62 ± 3,670.20 | 70,099.63 ± 11,475.40 | 33,208.06 ± 14,217.17 | HI | Glutamate synthesis |
| K05916 | Nitric oxide dioxygenase [EC:1.14.12.17] | Nitrosative stress defense | 25719.54 ± 1319.03 | 30733.94 ± 3388.51 | 26125.75 ± 420.66 | HI | Protects against nitrosative stress by converting NO to nitrate |
| K01915 | Glutamine synthetase [EC:6.3.1.2] | Nitrogen assimilation | 47130.22 ± 3501.23 | 57586.57 ± 8465.33 | 26414.7 ± 6455.3 | HI | Glutamine synthetase - nitrogen assimilation, stress response |
| K00382 | Dihydrolipoamide dehydrogenase [EC:1.8.1.4] | Redox homeostasis | 54090.06 ± 3491.25 | 105283.57 ± 11804.19 | 71617.49 ± 10418.09 | HI | Dihydrolipoamide dehydrogenase - redox homeostasis |
| K00384 | Thioredoxin reductase (NADPH) [EC:1.8.1.9] | Redox homeostasis | 77747.30 ± 5933.56 | 71908.45 ± 5443.11 | 83613.82 ± 671.74 | TI | Reduces oxidized thioredoxin, maintaining cellular redox balance |

**Note:** Data are mean ± SD (n = 3 replicate ponds per treatment: WS1–WS3, HS1–HS3, TS1–TS3). Different superscript letters in the same column denote statistically significant differences (*p* < 0.05), while identical letters indicate no significant differences (*p* > 0.05). Abbreviations: WI, HI, and TI denote the intestinal samples from groups W, H, and T, respectively.

**Supplementary Table S5 (continued).** KEGG-based annotation of immunity-related pathways and enzymes in intestinal microbiota across treatments W, H, and T (continued)

| **KO ID** | **KO Description** | **Pathway Category** | **WI** | **HI** | **TI** | **Dominent group** | **Functional Role** |
| --- | --- | --- | --- | --- | --- | --- | --- |
| K04564 | Superoxide dismutase, Fe-Mn family [EC:1.15.1.1] | Oxidative stress defense | 44668.36 ± 2840.18 | 55462.78 ± 6231.89 | 64639.24 ± 2540.07 | TI | Superoxide dismutase (Fe-Mn) - detoxifies superoxide radicals |
| K04565 | Superoxide dismutase, Cu-Zn family [EC:1.15.1.1] | Oxidative stress defense | 3384.78 ± 713.38 | 21970.15 ± 2459.92 | 26066.02 ± 6242.96 | TI | Superoxide dismutase (Cu-Zn) - detoxifies superoxide radicals |
| K03386 | Peroxiredoxin (alkyl hydroperoxide reductase subunit C) [EC:1.11.1.15] | Oxidative stress defense | 40329.77 ± 3052.88 | 28546.41 ± 1650.34 | 26225.88 ± 6156.35 | WI | Peroxiredoxin - reduces organic hydroperoxides and H₂O₂ |
| K03781 | Catalase [EC:1.11.1.6] | Oxidative stress defense | 6826.21 ± 1497.97 | 37876.46 ± 13617.45 | 64524.18 ± 2500.78 | TI | Catalase - decomposes hydrogen peroxide |
| K03782 | Catalase-peroxidase [EC:1.11.1.21] | Oxidative stress defense | 2363.59 ± 997.66 | 13273.1 ± 5953.19 | 6914.64 ± 7983.58 | HI | Catalase-peroxidase - broad-spectrum peroxide detoxification |
| K00432 | Glutathione peroxidase [EC:1.11.1.9] | Oxidative stress defense | 41557.1 ± 2973.03 | 34599.45 ± 1242.73 | 26425.68 ± 6306.38 | WI | Glutathione peroxidase - reduces organic hydroperoxides |
| K03387 | Alkyl hydroperoxide reductase subunit F [EC:1.6.4.-] | Oxidative stress defense | 35920.66 ± 3878.54 | 3118.39 ± 1521.58 | 26221.38 ± 6214.12 | WI | Alkyl hydroperoxide reductase - reduces organic peroxides |
| K11065 | Thiol peroxidase, atypical 2-Cys peroxiredoxin [EC:1.11.1.15] | Oxidative stress defense | 600.31 ± 411.68 | 3548.83 ± 2691.94 | 19188.44 ± 1890.68 | TI | Thiol peroxidase - atypical peroxiredoxin |

**Note:** Data are mean ± SD (n = 3 replicate ponds per treatment: WS1–WS3, HS1–HS3, TS1–TS3). Different superscript letters in the same column denote statistically significant differences (*p* < 0.05), while identical letters indicate no significant differences (*p* > 0.05). Abbreviations: WI, HI, and TI denote the intestinal samples from groups W, H, and T, respectively.

**Supplementary Table S5 (continued).** KEGG-based annotation of immunity-related pathways and enzymes in intestinal microbiota across treatments W, H, and T

| **KO ID** | **KO Description** | **Pathway Category** | **WI** | **HI** | **TI** | **Dominent group** | **Functional Role** |
| --- | --- | --- | --- | --- | --- | --- | --- |
| K04771 | Serine protease Do [EC:3.4.21.107] | Stress response | 43558.3 ± 2982.76 | 44864.1 ± 3172.85 | 26149.71 ± 6215.47 | HI | Serine protease - degrades misfolded proteins under stress |
| K04077 | Chaperonin GroEL | Stress response | 43710.63 ± 2934.21 | 45811.77 ± 4402.28 | 26240.8 ± 6145.05 | HI | Chaperonin - protein folding under stress |
| K04078 | Chaperonin GroES | Stress response | 43478.79 ± 2964.71 | 44938.74 ± 3978.63 | 26232.3 ± 6145.15 | HI | Chaperonin - protein folding under stress |
| K03671 | Thioredoxin 1 | Stress response | 41906.68 ± 3018.2 | 36156.53 ± 2730.54 | 26231.04 ± 6175.59 | WI | Thioredoxin - maintains cellular redox balance |
| K03672 | Thioredoxin 2 [EC:1.8.1.8] | Stress response | 4880.60 ± 340.31 | 12677.28 ± 1685.87 | 11935.59 ± 393.74 | HI | Involved in redox regulation and oxidative stress defense |

**Note:** Data are mean ± SD (n = 3 replicate ponds per treatment: WS1–WS3, HS1–HS3, TS1–TS3). Different superscript letters in the same column denote statistically significant differences (*p* < 0.05), while identical letters indicate no significant differences (*p* > 0.05). Abbreviations: WI, HI, and TI denote the intestinal samples from groups W, H, and T, respectively.

**Supplementary Table S6.** KEGG-based annotation of toxin-related (virulence-associated) genes in intestinal microbiota across treatments W, H, and T

| **KO ID** | **KO Description** | **WI** | **HI** | **TI** | **Dominent group** | **Functional Role** |
| --- | --- | --- | --- | --- | --- | --- |
| K11031 | Thiol-activated cytolysin | 37.51 ± 56.51 | 909.01 ± 1569.92 | 19115.88 ± 1826.12 | TI | Pore-forming toxin; disrupts cell membranes |

**Note:** Data are mean ± SD (n = 3 replicate ponds per treatment: WS1–WS3, HS1–HS3, TS1–TS3). Different superscript letters in the same column denote statistically significant differences (*p* < 0.05), while identical letters indicate no significant differences (*p* > 0.05). Abbreviations: WI, HI, and TI denote the intestinal samples from groups W, H, and T, respectively.

**Supplementary Table S6 (continued).** KEGG-based annotation of toxin-related (virulence-associated) genes in intestinal microbiota across treatments W, H, and T

| **KO ID** | **KO Description** | **WI** | **HI** | **TI** | **Dominent group** | **Functional Role** |
| --- | --- | --- | --- | --- | --- | --- |
| K11032 | Hemolysin II | 37.51 ± 56.51 | 908.01 ± 1569.92 | 19114.38 ± 1826.12 | TI | Cytolytic toxin; lyses host and microbial cells |
| K11033 | Non-hemolytic enterotoxin A | 37.51 ± 56.51 | 913.34 ± 1569.92 | 19119.38 ± 1826.12 | TI | Enterotoxin; disrupts intestinal cells, competition |
| K11034 | Non-hemolytic enterotoxin B/C | 75.01 ± 113.03 | 1817.34 ± 3139.83 | 38238.75 ± 3652.23 | TI | Tripartite enterotoxin; pore formation and cytotoxicity |
| K11041 | Exfoliative toxin A/B | 35307.00 ± 4273.38 | 175.47 ± 273.92 | 0.03 ± 0.00 | WI | Serine protease toxin; disrupts epithelial integrity |
| K11068 | Hemolysin III | 42000.53 ± 3103.97 | 39513.41 ± 3428.94 | 38614.18 ± 3737.98 | WI | Membrane-damaging toxin; broad cytolytic activity |
| K11005 | Hemolysin A | 142.68 ± 197.61 | 774.00 ± 438.14 | 3.02 ± 4.22 | HI | Hemolytic toxin; contributes to microbial competition |
| K11020 | Exotoxin A | 45.80 ± 19.14 | 210.40 ± 48.63 | 1.45 ± 1.49 | HI | ADP-ribosylating toxin; inhibits protein synthesis |

**Note:** Data are mean ± SD (n = 3 replicate ponds per treatment: WS1–WS3, HS1–HS3, TS1–TS3). Different superscript letters in the same column denote statistically significant differences (*p* < 0.05), while identical letters indicate no significant differences (*p* > 0.05). Abbreviations: WI, HI, and TI denote the intestinal samples from groups W, H, and T, respectively.

**Supplementary Table S7.** KEGG-based annotation of adhesion, colonization, and secretion system-related genes in intestinal microbiota across treatments W, H, and T

| **KO ID** | **Description** | **WI** | **HI** | **TI** |
| --- | --- | --- | --- | --- |
| K02650 | type IV pilus assembly protein PilA | 1612.5 ± 3063.0 | 6736.7 ± 7288.7 | 13617.0 ± 16325.6 |
| K02655 | type IV pilus assembly protein PilE | 912.1 ± 1152.1 | 5759.0 ± 7928.4 | 13739.8 ± 16270.2 |
| K02651 | pilus assembly protein Flp/PilA | 3533.9 ± 1127.3 | 18049.6 ± 2843.1 | 160.6 ± 65.0 |

**Note:** Data are mean ± SD (n = 3 replicate ponds per treatment: WS1–WS3, HS1–HS3, TS1–TS3). Different superscript letters in the same column denote statistically significant differences (*p* < 0.05), while identical letters indicate no significant differences (*p* > 0.05). Abbreviations: WI, HI, and TI denote the intestinal samples from groups W, H, and T, respectively.

**Supplementary Table S7 (continued).** KEGG-based annotation of adhesion, colonization, and secretion system-related genes in intestinal microbiota across treatments W, H, and T

| KO ID | Description | WI | HI | TI |
| --- | --- | --- | --- | --- |
| K03200 | type IV secretion system protein VirB5 | 421.2 ± 396.8 | 1247.1 ± 781.7 | 500.1 ± 693.9 |
| K02652 | type IV pilus assembly protein PilB | 408.1 ± 655.0 | 3008.9 ± 3020.8 | 6928.1 ± 8089.1 |
| K03204 | type IV secretion system protein VirB9 | 435.3 ± 428.7 | 1243.8 ± 779.1 | 5.2 ± 7.3 |
| K03205 | type IV secretion system protein VirD4 [EC:7.4.2.8] | 434.3 ± 554.8 | 1580.5 ± 1186.6 | 28.2 ± 29.9 |
| K02653 | type IV pilus assembly protein PilC | 454.9 ± 664.6 | 3045.2 ± 3108.7 | 733.4 ± 863.6 |
| K03196 | type IV secretion system protein VirB11 [EC:7.4.2.8] | 459.8 ± 564.8 | 1386.0 ± 915.5 | 5.2 ± 7.3 |
| K03219 | type III secretion protein C | 440.8 ± 542.3 | 1880.3 ± 820.5 | 13.5 ± 1.5 |

**Note:** Data are mean ± SD (n = 3 replicate ponds per treatment: WS1–WS3, HS1–HS3, TS1–TS3). Different superscript letters in the same column denote statistically significant differences (*p* < 0.05), while identical letters indicate no significant differences (*p* > 0.05). Abbreviations: WI, HI, and TI denote the intestinal samples from groups W, H, and T, respectively.

**Supplementary Table S8.** Post-mortem pH changes of shrimp meat (0–24 h) across treatments W, H, and T

| **Time (h)** | **WM (Control)** | **HM (Single Probiotic)** | **TM (Mixed Probiotic)** |
| --- | --- | --- | --- |
| 0 | 7.20 ± 0.02ᵇ | 7.23 ± 0.01ᵃᵇ | 7.24 ± 0.01ᵃ |
| 6 | 6.84 ± 0.13ᶜ | 6.94 ± 0.06ᵇ | 7.01 ± 0.04ᵃ |
| 12 | 6.42 ± 0.20ᶜ | 6.56 ± 0.08ᵇ | 6.79 ± 0.06ᵃ |
| 18 | 6.16 ± 0.22ᶜ | 6.30 ± 0.04ᵇ | 6.53 ± 0.10ᵃ |
| 24 | 5.91 ± 0.19ᶜ | 6.07 ± 0.05ᵇ | 6.10 ± 0.02ᵃ |

**Note:** Data are mean ± SD (n = 3 replicate ponds per treatment: WS1–WS3, HS1–HS3, TS1–TS3). Different superscript letters in the same column denote statistically significant differences (*p* < 0.05), while identical letters indicate no significant differences (*p* > 0.05). Abbreviations: WM, HM, and TM denote the meat samples from groups W, H, and T, respectively.

**Supplementary Table S9.** Full protein functional annotation table

| **Annotation database** | **Number of comments** | **Total number of proteins** | **Annotation rate** | **e_value** |
| --- | --- | --- | --- | --- |
| KEGG | 595 | 749 | 0.7948 | 0.00001 |
| NR | 748 | 749 | 0.9991 | - |
| EggNOG | 701 | 749 | 0.9362 | 0.00001 |
| Sub Cell-Location | 749 | 749 | 1 | - |
| Pfam | 690 | 749 | 0.9223 | 0.00001 |
| GO | 622 | 749 | 0.8306 | 0.00001 |

**Supplementary Table S10**. Statistical table of the number of differential proteins in shrimp meat across treatments W, H, and T

| **Differential group** | **Total protein** | **Up** | **Down** |
| --- | --- | --- | --- |
| HM vs. WM | 176 | 107 | 69 |
| TM vs. WM | 220 | 96 | 124 |
| HM vs. TM | 183 | 110 | 73 |

**Note:** Data are mean ± SD (n = 3 replicate ponds per treatment: WS1–WS3, HS1–HS3, TS1–TS3). Different superscript letters in the same column denote statistically significant differences (*p* < 0.05), while identical letters indicate no significant differences (*p* > 0.05). Abbreviations: WM, HM, and TM denote the meat samples from groups W, H, and T, respectively.

**Supplementary Table S11.** Protein abundance of major structural, calcium‑binding, and oxygen‑transport proteins in shrimp meat across group W, H, and T

| **Protein Family** | **Accession ID** | **Protein Name** | **WM** | **HM** | **TM** | **Functional Role** |
| --- | --- | --- | --- | --- | --- | --- |
| Myosin heavy chain | A0A3R7PJ50 | Myosin heavy chain type 2 (Fragment) | 2066.88 ± 326.58 | 3028.79 ± 335.72 | 2166.80 ± 336.44 | Major contractile protein; determines muscle fiber structure and texture |
|  | A0A3R7Q491 | Myosin heavy chain type b | 1930.74 ± 1218.00 | 2179.60 ± 527.66 | 2736.66 ± 543.08 | Contractile function; influences meat firmness |
|  | A0A3R7SLR1 | Myosin heavy chain type 1 | 683.24 ± 71.03 | 1276.29 ± 1155.25 | 962.93 ± 373.99 | Contractile function |

**Note:** Data are mean ± SD (n = 3 replicate ponds per treatment: WS1–WS3, HS1–HS3, TS1–TS3). Different superscript letters in the same column denote statistically significant differences (*p* < 0.05), while identical letters indicate no significant differences (*p* > 0.05). Abbreviations: WM, HM, and TM denote the meat samples from groups W, H, and T, respectively.

**Supplementary Table S11 (continued).** Protein abundance of major structural, calcium‑binding, and oxygen‑transport proteins in shrimp meat across group W, H, and T

| **Protein Family** | **Accession ID** | **Protein Name** | **WM** | **HM** | **TM** | **Functional Role** |
| --- | --- | --- | --- | --- | --- | --- |
| Myosin heavy chain | K4Q2S1 | Myosin heavy chain type 2 | 30770.54 ± 432.83 | 30285.15 ± 1548.21 | 31038.21 ± 458.55 | Major myosin isoform in muscle |
|  | N0DTS3 | Myosin heavy chain type 4 (Fragment) | 136.40 ± 81.78 | 345.57 ± 394.26 | 93.78 ± 62.05 | Fast-twitch muscle fiber component |
|  | N0DTS9 | Myosin heavy chain type 6a (Fragment) | 220.97 ± 103.78 | 287.71 ± 262.19 | 138.16 ± 91.45 | Cardiac/slow muscle isoform |
| Myosin light chain | A0A3R7N886 | Myosin essential light chain | 81.47 ± 24.15 | 160.58 ± 58.11 | 131.13 ± 77.25 | Regulates myosin ATPase activity and muscle contraction |
|  | A0A3R7PTG0 | Myosin light chain 2 | 23509.71 ± 4100.60 | 13620.80 ± 6708.42 | 31351.09 ± 18745.68 | Structural and regulatory function |
|  | B7SNI3 | Myosin regulatory light chain 2 | 347.62 ± 99.13 | 442.75 ± 141.20 | 533.31 ± 40.60 | Regulates muscle contraction via phosphorylation |
| Actin | A0A3R7Q5M1 | Actin, muscle | 4113.48 ± 426.15 | 4352.25 ± 27.52 | 2318.90 ± 2325.12 | Major cytoskeletal protein; forms thin filaments |
|  | A0A3R7SP81 | Actin 1 | 152.72 ± 24.47 | 277.62 ± 114.31 | 321.23 ± 96.13 | Cytoskeletal structure |
|  | A0A423SPG0 | Actin 2 | 9659.54 ± 440.18 | 8638.94 ± 719.39 | 9861.59 ± 957.70 | Cytoskeletal structure |
|  | Q9GSP9 | Beta-actin | 18773.07 ± 3119.65 | 16983.22 ± 3483.22 | 17825.95 ± 3881.87 | Ubiquitous actin isoform |
|  | A0A3R7T7C2 | Skeletal muscle actin 6 | 4704.27 ± 340.15 | 4750.59 ± 308.28 | 4791.85 ± 128.69 | Muscle-specific actin |
| Calcium-binding proteins | A0A3R7NZC3 | Sarcoplasmic calcium-binding protein variant a | 2492.12 ± 523.54 | 3202.36 ± 74.66 | 3236.20 ± 519.04 | Regulates calcium signaling and muscle contraction |
|  | A0A423SPI4 | Sarcoplasmic calcium-binding protein | 3007.75 ± 1829.54 | 1140.90 ± 4.43 | 1133.26 ± 10.95 | Calcium buffering in sarcoplasm |
|  | A0A423T8H7 | Sarcoplasmic calcium-binding protein, beta chain | 105344.42 ± 5472.19 | 80655.91 ± 25109.68 | 100640.98 ± 3978.17 | Calcium regulation |
| Collagen | A0A423SAL8 | Collagen alpha-1(V) chain | 133.0536016 | 168.2007192 | 194.1136271 | Protein digestion and absorption |

**Note:** Data are mean ± SD (n = 3 replicate ponds per treatment: WS1–WS3, HS1–HS3, TS1–TS3). Different superscript letters in the same column denote statistically significant differences (*p* < 0.05), while identical letters indicate no significant differences (*p* > 0.05). Abbreviations: WM, HM, and TM denote the meat samples from groups W, H, and T, respectively.

**Supplementary Table S11 (continued).** Protein abundance of major structural, calcium‑binding, and oxygen‑transport proteins in shrimp meat across group W, H, and T

| **Protein Family** | **Accession ID** | **Protein Name** | **WM** | **HM** | **TM** | **Functional Role** |
| --- | --- | --- | --- | --- | --- | --- |
| Hemocyanin (oxygen transport) | A0A059TEW9 | Hemocyanin subunit L1 (Fragment) | 3077.67 ± 146.74 | 3509.24 ± 794.15 | 4285.86 ± 45.18 | Oxygen transport; may affect color stability |
|  | A0A059TIS0 | Hemocyanin subunit L5 (Fragment) | 283.43 ± 73.26 | 616.46 ± 57.65 | 370.19 ± 15.58 | Oxygen transport |
|  | A0A3R7LSN5 | Hemocyanin subunit L1 | 206.17 ± 94.46 | 604.61 ± 219.22 | 341.11 ± 22.70 | Oxygen transport |
|  | A0A423SGU8 | Hemocyanin subunit L2 | 2539.99 ± 211.17 | 5930.95 ± 2237.58 | 4284.26 ± 1931.54 | Oxygen transport |
|  | Q26180 | Hemocyanin | 5686.51 ± 118.55 | 7354.73 ± 385.66 | 8802.96 ± 272.73 | Major oxygen carrier in crustaceans |

**Note:** Data are mean ± SD (n = 3 replicate ponds per treatment: WS1–WS3, HS1–HS3, TS1–TS3). Different superscript letters in the same column denote statistically significant differences (*p* < 0.05), while identical letters indicate no significant differences (*p* > 0.05). Abbreviations: WM, HM, and TM denote the meat samples from groups W, H, and T, respectively.

**Supplementary Table S12.** Differential expression of carbohydrate metabolism enzymes and pathways in shrimp muscle across group W, H, and T

| **Accession ID** | **Protein Name** | **Symbol** | **Pathway** | **HM** | **WM** | **TM** |
| --- | --- | --- | --- | --- | --- | --- |
| A0A2S1P7N3 | Glyceraldehyde-3-phosphate dehydrogenase | GAPDH | Glycolysis | 36344 ± 2788 | 39397 ± 3263 | 27497 ± 2642 |
| K0E682 | Triosephosphate isomerase | TPI | Glycolysis | 11492 ± 2267 | 15651 ± 432 | 12482 ± 4050 |
| A0A4Y5R070 | Fructose-bisphosphate aldolase | FBA | Glycolysis | 5323 ± 492 | 7560 ± 613 | 3914 ± 1644 |
| A0A3R7N0A1 | Phosphoglucomutase | PGM | Glycolysis | 2025 ± 357 | 1831 ± 446 | 1079 ± 58 |
| A0A3R7PAZ3 | Glycerol-3-phosphate dehydrogenase [NAD(+)] | GPD1 | Glycolysis | 1486 ± 248 | 1525 ± 296 | 1049 ± 280 |
| A0A3R7MD67 | Glycogen debranching enzyme | AGL | Starch metabolism | 968 ± 128 | 971 ± 113 | 734 ± 128 |

**Note:** Data are mean ± SD (n = 3 replicate ponds per treatment: WS1–WS3, HS1–HS3, TS1–TS3). Different superscript letters in the same column denote statistically significant differences (*p* < 0.05), while identical letters indicate no significant differences (*p* > 0.05). Abbreviations: WM, HM, and TM denote the meat samples from groups W, H, and T, respectively.

**Supplementary Table S12 (continued).** Differential expression of carbohydrate metabolism enzymes and pathways in shrimp muscle across group W, H, and T

| **Accession ID** | **Protein Name** | **Symbol** | **Pathway** | **HM** | **WM** | **TM** |
| --- | --- | --- | --- | --- | --- | --- |
| A0A423T8A3 | Phosphorylase b kinase regulatory subunit | PHKA_B | Signaling | 289 ± 60 | 443 ± 20 | 291 ± 61 |
| A0A3R7QLT7 | Polysaccharide lyase family protein | - | - | 332 ± 169 | 90 ± 4.5 | 70 ± 20.9 |
| A0A3R7M7J0 | Phosphorylase kinase subunit gamma | PHKG | Signaling | 148 ± 14 | 145 ± 18 | 105 ± 27 |
| A0A3R7PWQ4 | 1,4-alpha-glucan branching enzyme | GBE1 | Starch metabolism | 116 ± 22 | 122 ± 13 | 88 ± 18 |
| I1SSL2 | L-lactate dehydrogenase | (L-LDH) | Pyruvate metabolism, Glycolysis/Gluconeogenesis | 2616.89 ± 56.05 | 5301.55 ± 105.6 | 2166.21 ± 2.10 |
| I1VSB4 | L-lactate dehydrogenase (L-LDH) | (L-LDH) | Pyruvate metabolism, Glycolysis/Gluconeogenesis | 444.99 ± 25.76 | 1096.17 ± 126.03 | 429.82 ± 1.58 |

**Note:** Data are mean ± SD (n = 3 replicate ponds per treatment: WS1–WS3, HS1–HS3, TS1–TS3). Different superscript letters in the same column denote statistically significant differences (*p* < 0.05), while identical letters indicate no significant differences (*p* > 0.05). Abbreviations: WM, HM, and TM denote the meat samples from groups W, H, and T, respectively.

**Supplementary Table S13.** Differential expression of proteolytic enzyms in shrimp meat across group W, H, and T

| **Accession ID** | **Protein name** | **KO ID** | **Pathway ID** | **Pathway** | **HM** | **WM** | **TM** |
| --- | --- | --- | --- | --- | --- | --- | --- |
| Q00871 | Chymotrypsin BII | — | — | — | 163.50±86.8 | 225.39±73.6 | 129.80±20.6 |
| A0A423TZK7 | Carboxypeptidase A1 | K08779; K01298 | map0497; map04974 | Protein digestion & absorption | 210.4±49.4 | 145.7±20.3 | 94.5±27.3 |
| A0A423SQ82 | Calpain M | K08585 | — | — | 109.15±20.2 | 147.68±12.2 | 156.51±0.83 |

**Note:** Data are mean ± SD (n = 3 replicate ponds per treatment: WS1–WS3, HS1–HS3, TS1–TS3). Different superscript letters in the same column denote statistically significant differences (*p* < 0.05), while identical letters indicate no significant differences (*p* > 0.05). Abbreviations: WM, HM, and TM denote the meat samples from groups W, H, and T, respectively.

**Supplementary Table S13 (continued).** Differential expression of proteolytic enzyms in shrimp meat across group W, H, and T

| **Accession ID** | **Protein name** | **KO ID** | **Pathway ID** | **Pathway** | **HM** | **WM** | **TM** |
| --- | --- | --- | --- | --- | --- | --- | --- |
| A0A423TPX1 | Metalloproteinase domain protein | K06704 | map0501; map0512 | Signaling & regeneration | 40.8±47.2 | 85.8±110.3 | 80.3±4.8 |
| Q20AS8 | Carboxypeptidase B | K01298 | map04974 | Protein digestion | 106.0±35.1 | 132.5±43.7 | 52.1±27.2 |
| A0A3R7ST81 | Cathepsin L | K01365 | map04140 | Lysosome/autophagy | 72.7±15.3 | 103.8±12.9 | 46.7±5.2 |
| A0A3R7P4C8 | Proteasome subunit beta | K02738 | map0305 | Proteasome | 37.6±3.9 | 57.9±13.5 | 71.5±16.7 |
| A0A423U9K3 | Carboxypeptidase A2-like | — | — | Protein digestion | 126.0±46.7 | 39.7±1.0 | 20.7±11.7 |
| A0A423SFG7 | Dipeptidyl peptidase 1 | K01275 | map0421 | Apoptosis | 93.6±44.3 | 22.2±2.9 | 38.0±12.2 |
| A0A423ST31 | Aminopeptidase | K11140 | map0048 | Glutathione metabolism | 13.2±5.3 | 13.5±1.8 | 8.9±3.0 |

**Note:** Data are mean ± SD (n = 3 replicate ponds per treatment: WS1–WS3, HS1–HS3, TS1–TS3). Different superscript letters in the same column denote statistically significant differences (*p* < 0.05), while identical letters indicate no significant differences (*p* > 0.05). Abbreviations: WM, HM, and TM denote the meat samples from groups W, H, and T, respectively.

**Supplementary Table S14.** Differential expression of lipid metabolism enzymes in shrimp meat across group W, H, and T

| **Accession ID** | **Protein name** | **KO ID** | **Pathway ID** | **Pathway** | **WM** | **HM** | **TM** |
| --- | --- | --- | --- | --- | --- | --- | --- |
| A0A3R7QAN5 | Hydroxyacyl-coenzyme A dehydrogenase, mitochondrial | K00022 | map00062, map00071 | Fatty acid elongation; Fatty acid degradation | 42.22 ± 10.12 | 132.53 ± 28.58 | 43.00 ± 14.17 |
| A0A3R7SLU0 | Phospholipase D | K01115 | map04666, map04072, map04071, | Glycerophospholipid metabolism; Phospholipase D signaling pathway; Sphingolipid signaling pathway; | 144.79 ± 18.78 | 97.03 ± 13.48 | 120.39 ± 4.26 |
| A0A3R7QP52 | Long-chain-fatty-acid--CoA ligase | K01897 | map04714, map00061, map00071, map04146 | Thermogenesis; Fatty acid biosynthesis; Fatty acid degradation | 48.97 ± 5.97 | 59.52 ± 8.38 | 50.18 ± 12.19 |

**Note:** Data are mean ± SD (n = 3 replicate ponds per treatment: WS1–WS3, HS1–HS3, TS1–TS3). Different superscript letters in the same column denote statistically significant differences (*p* < 0.05), while identical letters indicate no significant differences (*p* > 0.05). Abbreviations: WM, HM, and TM denote the meat samples from groups W, H, and T, respectively.

**Supplementary Table S14 (continued).** Differential expression of lipid metabolism enzymes in shrimp meat across group W, H, and T

| **Accession ID** | **Protein name** | **KO ID** | **Pathway ID** | **Pathway** | **WM** | **HM** | **TM** |
| --- | --- | --- | --- | --- | --- | --- | --- |
| A0A3R7PGB4 | 3-ketoacyl-CoA thiolase, mitochondrial | K07508 | map00071, map00062 | Fatty acid degradation; Fatty acid elongation | 79.15 ± 20.94 | 56.51 ± 8.43 | 64.77 ± 14.94 |
| A0A423TY88 | Medium-chain specific acyl-CoA dehydrogenase, mitochondrial | K00249 | map00071 | Fatty acid degradation | 24.52 ± 0.00 | 55.71 ± 1.00 | 20.37 ± 0.00 |
| A0A423UAL3 | Phosphoinositide phospholipase C | K05857 | map04020, | Calcium signaling pathway; | 42.48 ± 9.12 | 48.30 ± 3.33 | 45.37 ± 6.09 |
| A0A3R7PAZ3 | Glycerol-3-phosphate dehydrogenase [NAD(+)] | K00006 | map00564 | Glycerophospholipid metabolism | 1525 ± 296 | 1486 ± 248 | 1049 ± 280 |

**Note:** Data are mean ± SD (n = 3 replicate ponds per treatment: WS1–WS3, HS1–HS3, TS1–TS3). Different superscript letters in the same column denote statistically significant differences (*p* < 0.05), while identical letters indicate no significant differences (*p* > 0.05). Abbreviations: WM, HM, and TM denote the meat samples from groups W, H, and T, respectively.

**Supplementary Table S15.** Differential expression of antioxidant and stress-related proteins in shrimp meat across group W, H, and T

| **Accession ID** | **Full Protein Name** | **Short Name** | **Function** | **WM** | **HM** | **TM** |
| --- | --- | --- | --- | --- | --- | --- |
| A0A3R7QHC4 | Catalase | CAT | Antioxidant | 29.41 ± 24.70 | 33.40 ± 20.31 | 26.11 ± 24.57 |
| D0FH89 | Peroxiredoxin | PRDX | Antioxidant | 36.63 ± 3.50 | 46.80 ± 13.67 | 65.93 ± 17.89 |
| S4VEU6 | Manganese superoxide dismutase 2 | MnSOD2 | Antioxidant | 88.16 ± 54.94 | 147.44 ± 29.80 | 120.29 ± 36.66 |
| A0A423SGR7 | Peroxiredoxin 6 | PRDX6 | Antioxidant | 32.30 ± 1.73 | 48.74 ± 4.31 | 82.56 ± 18.07 |
| A0A3R7SMK3 | Glutathione S-transferase | GST | Detoxification | 355.95 ± 82.60 | 530.89 ± 44.63 | 634.13 ± 148.16 |
| A0A423SAG9 | Cathepsin D | CTSD (S1) | Lysosome | 23.19 ± 5.59 | 5.90 ± 10.22 | 29.62 ± 8.59 |
| A0A3R7SR07 | Cathepsin D | CTSD (S2) | Lysosome | 48.67 ± 7.95 | 46.01 ± 3.96 | 70.24 ± 17.88 |

**Note:** Data are mean ± SD (n = 3 replicate ponds per treatment: WS1–WS3, HS1–HS3, TS1–TS3). Different superscript letters in the same column denote statistically significant differences (*p* < 0.05), while identical letters indicate no significant differences (*p* > 0.05). Abbreviations: WM, HM, and TM denote the meat samples from groups W, H, and T, respectively.

**Supplementary Table S15 (continued).** Differential expression of antioxidant and stress-related proteins in shrimp meat across group W, H, and T

| **Accession ID** | **Full Protein Name** | **Short Name** | **Function** | **WM** | **HM** | **TM** |
| --- | --- | --- | --- | --- | --- | --- |
| A0A3R7MS79 | Heat shock protein 60 | HSP60 | Stress protein | 127.62 ± 52.54 | 81.99 ± 15.00 | 81.65 ± 11.01 |
| A0A3R7P9A3 | Heat shock protein 70 | HSP70 | Stress protein | 48.64 ± 13.62 | 26.59 ± 5.92 | 51.37 ± 25.95 |
| A0A3R7PLZ1 | Selenium-binding protein | SBP | Metal regulation | 19.00 ± 4.00 | 29.00 ± 5.00 | 27.00 ± 3.00 |

**Note:** Data are mean ± SD (n = 3 replicate ponds per treatment: WS1–WS3, HS1–HS3, TS1–TS3). Different superscript letters in the same column denote statistically significant differences (*p* < 0.05), while identical letters indicate no significant differences (*p* > 0.05). Abbreviations: WM, HM, and TM denote the meat samples from groups W, H, and T, respectively.

**Supplementary Table S16.** Differential expression of mineral absorption / uptake / transport related proteins in shrimp meat across group W, H, and T

| **Protein Name** | **KO ID** | **Pathway ID** | **Pathway Definition** | **WM** | **HM** | **TM** |
| --- | --- | --- | --- | --- | --- | --- |
| Metallothionein | K14741 | – | – | 0.00 ± 0.00 | 8.76 ± 0.96 | 5.87 ± 0.89 |
| Sodium/potassium-transporting ATPase subunit alpha | K01539 | map04978, map04976, map04973, map04911 | Mineral absorption, Bile secretion, Carbohydrate digestion and absorption, Insulin secretion, Salivary secretion | 574.44± 93.41 | 618.88 ± 30.09 | 483.05± 75.19 |
| Sodium/potassium-transporting ATPase subunit beta | K01540 | map04978, map04976, map04973, map04911 | Mineral absorption, Bile secretion, Carbohydrate digestion and absorption, Insulin secretion, Salivary secretion | 85.01± 7.03 | 91.36± 14.78 | 86.90± 23.27 |
| Sodium/calcium exchanger 1 | K05849 | map04978, map04961, map04974, map04020 | Mineral absorption, Endocrine and other factor-regulated calcium reabsorption, Protein digestion and absorption, Calcium signaling pathway | 891.29± 42.26 | 1407.53± 191.71 | 1088.79± 195.71 |

**Note:** Data are mean ± SD (n = 3 replicate ponds per treatment: WS1–WS3, HS1–HS3, TS1–TS3). Different superscript letters in the same column denote statistically significant differences (*p* < 0.05), while identical letters indicate no significant differences (*p* > 0.05). Abbreviations: WM, HM, and TM denote the meat samples from groups W, H, and T, respectively.

**Supplementary Table S16 (continued).** Differential expression of mineral absorption / uptake / transport related proteins in shrimp meat across group W, H, and T

| **Protein Name** | **KO ID** | **Pathway ID** | **Pathway Definition** | **WM** | **HM** | **TM** |
| --- | --- | --- | --- | --- | --- | --- |
| Sarcoplasmic calcium-binding protein variant a | – | – | – | 100595.58 ± 5449.44 | 80655.91 ± 16527.38 | 101303.06 ± 4294.46 |
| Calcium-transporting ATPase | K05853 | map04020 | Calcium signaling pathway | 10937.99 ± 295.01 | 9652.43 ± 315.93 | 8119.98 ± 302.95 |
| Troponin C (Fragment) | – | – | – | 8354.30 ± 917.23 | 6118.46 ± 2610.62 | 7020.98 ± 2810.30 |
| Calmodulin | K02183 |  |  | 585.59 ± 19.58 | 491.19 ± 36.77 | 451.37 ± 67.24 |
| Troponin C1 | – | – | – | 500.86 ± 196.46 | 632.05 ± 119.32 | 679.41 ± 136.95 |
| Troponin I | – | – | – | 14430.57 ± 679.47 | 15314.42 ± 5591.63 | 11795.02 ± 5448.12 |

**Note:** Data are mean ± SD (n = 3 replicate ponds per treatment: WS1–WS3, HS1–HS3, TS1–TS3). Different superscript letters in the same column denote statistically significant differences (*p* < 0.05), while identical letters indicate no significant differences (*p* > 0.05). Abbreviations: WM, HM, and TM denote the meat samples from groups W, H, and T, respectively.

**Supplementary Table S17.** Differential expression of major and minor allergenic protein in shrimp meat across group W, H, and T

| **Accession ID** | **Protein Name** | **WM** | **HM** | **TM** | **Dominant group** |
| --- | --- | --- | --- | --- | --- |
| B4YAH6 | Lit v 1 tropomyosin | 31547.36 ± 1679.19 | 29358.71 ± 640.41 | 29976.91 ± 1155.31 | WM |
| A0A3R7PEF6 | Arginine kinase | 23648.69 ± 1536.61 | 24342.25 ± 2383.17 | 22579.68 ± 1483.09 | HM |
| A0A423T8H7 | Sarcoplasmic calcium-binding protein, beta chain | 105344.42 ± 5472.19 | 80655.91 ± 25109.68 | 100640.98 ± 3978.17 | WM |
| A0A3R7NZC3 | Sarcoplasmic calcium-binding protein variant a | 4704.27 ± 340.15 | 4750.59 ± 308.28 | 4791.85 ± 128.69 | TI |
| A0A3R7PTG0 | Myosin light chain 2 | 23543.05 ± 4630.03 | 13620.82 ± 6639.35 | 31351.12 ± 18996.7 | TM |
